# Xenarthrans of the collection of Santiago Roth from the Pampean Region of Argentina (Pleistocene), in Zurich, Switzerland

**DOI:** 10.1186/s13358-023-00265-7

**Published:** 2023-03-28

**Authors:** Kévin Le Verger

**Affiliations:** Palaeontological Institute and Museum, University of Zurich, Karl-Schmid-Strasse 4, 8006 Zurich, Switzerland

**Keywords:** Cingulata, Pilosa, Taxonomy, Diversity, Paleoecology, Cingulata, Pilosa, Taxonomía, Diversidad, Paleoecología

## Abstract

The present work concerns xenarthrans from the collection of Santiago (Kaspar Jakob) Roth (1850–1924) housed at the Palaeontological Institute and Museum of the University of Zurich, one of the most important collections of Pleistocene mammals from Argentina in Europe. Roth was a paleontologist originally from Switzerland who prospected and collected a large amount of Pleistocene megafauna of the Pampean Region of Argentina. The xenarthrans are the main representatives of this collection in Zurich, with 150 specimens. Since 1920, this material has not been revised and is under studied. The present investigation corresponds to a taxonomic revision resulting in 114 reassignments, leading to document xenarthran diversity and discuss their paleoecologies. The high diversity reflects the paleoecology of the Pampean Region during the Pleistocene, with the various abiotic events that impacted the paleoenvironment of this region. Within the Cingulata, the Pampean Region fauna was probably dominated by glyptodonts with a high representation of Glyptodontinae and Neosclerocalyptinae while within the sloths the highest diversity and abundance is found in the Mylodontinae and Scelidotheriinae. These four clades represent both species with high ecological tolerance (e.g., *Glyptodon munizi*; *Catonyx tarijensis*) and ecologically highly specialized species (e.g., *Neosclerocalyptus paskoensis*; *Scelidotherium leptocephalum*). The presence of such ecological diversity underlines the status of the Pampean Region as a major interest for paleoecological and paleoenvironmental reconstruction.

**Supplementary Information:**

The online version contains supplementary material available at 10.1186/s13358-023-00265-7.

## Resumen

El presente trabajo se refiere a los xenartros de la colección de Santiago (Kaspar Jakob) Roth (1850-1924) depositada en el Instituto y Museo Paleontológico de la Universidad de Zurich, una de las más importantes colecciones de mamíferos del Pleistoceno de Argentina en Europa. Roth fue un paleontólogo originario de Suiza que prospectó y coleccionó una gran cantidad de megafauna del Pleistoceno de la Región Pampeana de Argentina. Los xenartros son los principales representantes de esta colección en Zurich, con 150 ejemplares. Desde 1920, este material no ha sido revisado y está poco estudiado. La presente investigación corresponde a una revisión taxonómica que ha dado lugar a 110 reasignaciones, lo que permite documentar la diversidad de los xenartros y discutir aspectos paleoecológicos. La alta diversidad refleja la paleoecología de la Región Pampeana durante el Pleistoceno en asociación con los cambios climáticos que han impactado en el paleoambiente de esta región. Entre los Cingulata, la fauna de la Región Pampeana estuvo probablemente dominada por los gliptodontes con una alta representación de Glyptodontinae y Neosclerocalyptinae, mientras que entre los perezosos la mayor diversidad y abundancia se encuentra en los Mylodontinae y Scelidotherinae. Estos cuatro clados representan tanto especies con alta tolerancia ecológica (por ejemplo, *Glyptodon munizi; Catonyx tarijensis*) como otras muy especializadas ecológicamente (por ejemplo, *Neosclerocalyptus paskoensis; Scelidotherium leptocephalum*). La presencia de tal diversidad ecológica subraya el estatus de la Región Pampeana como de gran interés para la reconstrucción paleoecológica y paleoambiental.

## Introduction

**Fig. 1:**
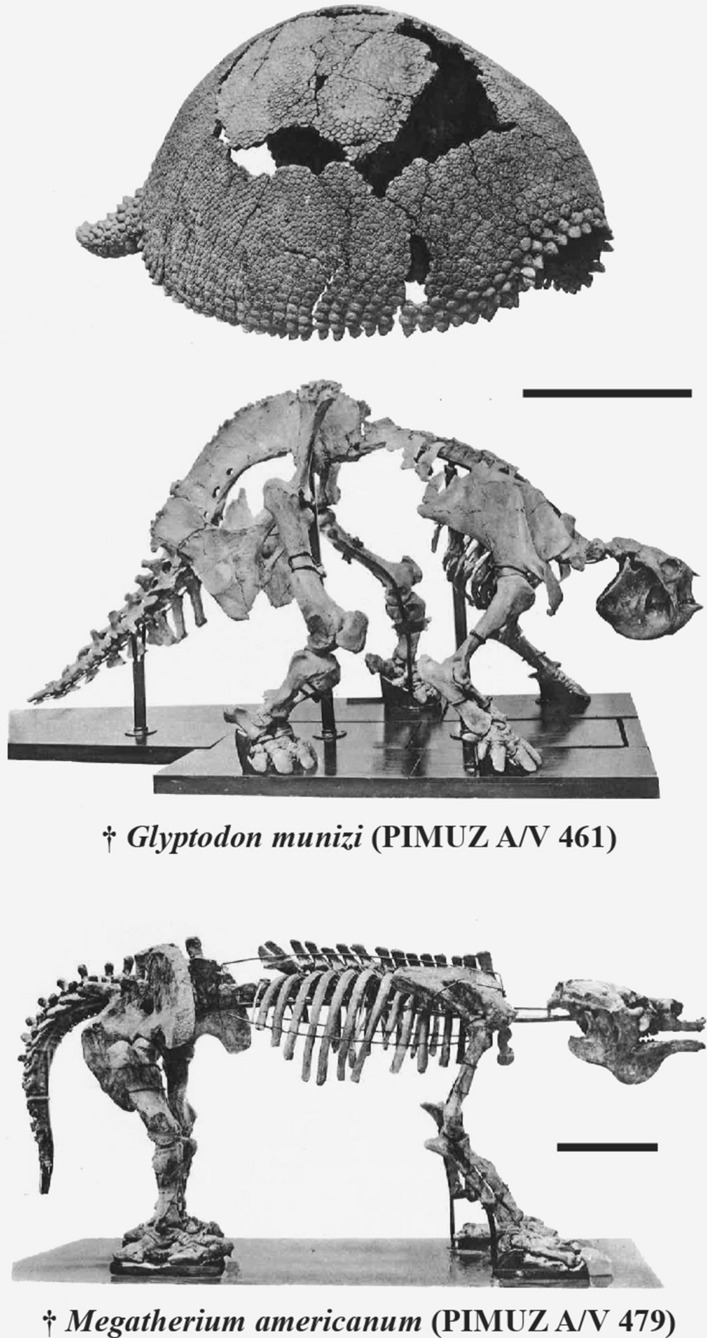
Xenarthran subcomplete specimens in right lateral view from the Roth collection at PIMUZ modified from Schulthess (1920). Above, *Glyptodon munizi* (PIMUZ A/V 461) associated with its carapace. Below, *Megatherium americanum* (PIMUZ A/V 479). Scale bar: 50 cm

Major advances in paleontology are achieved by discoveries made in collection from fossils long collected in the field. After more than 200 years of collecting around the world, some collections are now forgotten, lost, or understudied. In Europe, recently, several institutions are trying to highlight the most significant paleontological collection of mammals (e.g., Carrillo-Briceño et al., 2016; Solé et al., 2020; Van der Hoek, 2021; Vera et al., 2015; Zurita-Altamirano et al., 2019), a primacy for paleontological community. An example is the significant collection of Pleistocene megafauna from South America assembled by Santiago Roth. This Swiss-Argentinian paleontologist collected many fossil mammals during the second half of the nineteenth century (Sánchez-Villagra et al., in prep). Following numerous surveys, he sold a large part of his collection to institutions in Switzerland and Denmark. To date, the collection of Santiago Roth constitutes a significant part of the mammal collections of the Museo de la Plata in Argentina, the Zoological Museum of the University of Copenhagen in Denmark, and of the Natural History Museum of Geneva, the Cantonal Museum of Geology of the University of Lausanne, and the Palaeontological Institute and Museum of the University of Zurich (= PIMUZ) in Switzerland. In Europe, the collection of Santiago Roth gather many mammals belonging to the Pampean Region, a well-defined geographical area containing several geological formations covering a large part of the Pleistocene, from the end of the Sanandresian (ca. 2.0 Ma) to the end of the Lujanian (ca. 7.0 ka); according to Ages/Stages (see Cione & Tonni, 1995; Cione et al., 2015; Hansen, 2019; Prevosti & Forasiepi, 2018; Voglino, 2020). Many mammalian clades are represented, including the iconic toxodonts, gomphotheres, and macrauchenias (Roth, 1889). The collection of Santiago Roth stored in Europe mostly contains xenarthrans, represented by the giant glyptodonts (Cingulata) and ground sloths (Pilosa) (Roth, 1889) (Fig. 1).

Xenarthrans originated in South America (Vizcaíno & Loughry, 2008), with the earliest occurrence dated to approximately 55 Ma (Bergqvist et al., 2004; Gaudin & Croft, 2015) and a molecular estimate of their origin around the Cretaceous-Paleogene boundary (Delsuc et al., 2012; Gibb et al., 2016; Meredith et al., 2011). They underwent what George Gaylord Simpson called the "*Splendid Isolation*" (Simpson, 1980) until the full formation of the Isthmus of Panama, at the end of the Pliocene, although the date of closure is still debated, which led to the Great American Biotic Interchange (Domingo et al., 2020). Following this great event and in adequation with the great ice ages of the Quaternary, most of the South American megafauna increased size and diversity considerably before becoming extinct during the Holocene (Barnosky & Lindsey, 2010; Prado et al., 2015). Xenarthrans correspond to their most abundant and diversified representatives according to the Quaternary fossil record (see, for instance, Cione et al., 1999; Soibelzon et al., 2010; Vizcaíno & Loughry, 2008).

When Santiago Roth sold part of his collection to the canton of Zurich at the end of the nineteenth century, he published a list with short description of the specimens on his 5th catalog (Roth, 1889). Later, Schulthess (1920) taxonomically reassessed the xenarthrans from PIMUZ collections. Several specimens were later investigated by Guth (1961) for his study on the temporal region of edentates. De Iuliis (1996) included a ground sloth specimen from the collection of Roth at PIMUZ in his revision of the systematics of the Megatheriinae. During the last 20 years, with the exception of a study on the histology and bone compactness of xenarthran long bones (Straehl et al., 2013), PIMUZ specimens from the collection of Roth were sporadically integrated into much larger datasets for the postcranial anatomical description of *Eutatus* Gervais, 1867 (Krmpotic et al., 2009), for the revision of the systematics of Scelidotheriinae based on cranial materials (Corona et al., 2013; Miño-Boilini, 2012, 2016; Miño-Boilini & Quiñones, 2020; Miño-Boilini & Zurita, 2015; Miño-Boilini et al., 2014), for functional and ecomorphological studies using histology and microanatomy of several postcranial bones in mammals (Houssaye et al., 2016a) and on a larger evolutionary level (Houssaye et al., 2016b), and for the systematics of the Nothrotheriinae (Brandoni & Vezzosi, 2019; Vezzosi et al., 2019). It should be noted that since the work of Guth (1961), no study has incorporated glyptodonts from this collection, or even more broadly Cingulata with the exception of Krmpotic et al. (2009). Therefore, the xenarthrans of the Roth collection at PIMUZ, both armadillos and sloths, remain largely understudied and, since the work of Schulthess (1920), all of these specimens need to be reassessed in their taxonomy in light of recent work.

In the present study, xenarthran diversity of the Roth collection at PIMUZ was revised. First, I provide a list referring to the material of this collection at the species level associated with all the revised information for each specimen and comments when some singularity is to be specified. Concurrently, for each taxon, I refer to the completeness of the material particularly exceptional in this collection. Finally, I analyze the overall diversity in the context of the Pampean Region of the Pleistocene of Argentina. In addition, I briefly discuss several faunal assemblages resulting from the Roth xenarthrans at PIMUZ and highlight their potential implications for the paleoecology and paleoenvironment of the Pampean region during part of the Quaternary period.

## Materials and methods

The material analyzed corresponds to the xenarthrans from the 5th catalog named "*Fossiles de la Pampa*" provided with the specimens sold to the canton of Zurich by Santiago Roth (1889). All the pieces of the same individual were assigned to one number. I have revised each specimen in this catalog. Schulthess (1920) provided taxonomic assignments of each specimen. I reassessed these attributions with respect to the latest emended diagnosis of each species. In agreement with De Iuliis (2018), I favored reassignment to previously described species by pointing out peculiarities outside of the diagnoses rather than describing new species. The literature consulted was mentioned on a case-by-case basis which encouraged me to discuss the results in the same section. When a reassignment was necessary, I justified it for the specimen concerned. If a clear identification cannot be made, I have favored the use of an open taxonomy. The phylogeny used to discuss the diversity of Xenarthra from the Pampean Region is a concatenation of hypotheses presented in several articles (e.g., Boscaini et al., 2019; Defler, 2018; Núñez-Blasco et al., 2021; Presslee et al., 2019). Several phylogenies have been proposed based on morphological or molecular data (e.g., Delsuc et al., 2016; Gaudin & Lyon, 2017). In the present study, I follow Delsuc et al. (2016), who treat all families at the ‘subfamily’ rank level, except for Dasypodidae and Chlamyphoridae, and with the inclusion of glyptodonts within the latter clade. For Pilosa, I followed the dental nomenclature of Hautier et al. (2016); and the cranial description of Boscaini et al. (2020a). For Cingulata, the dental nomenclature is not defined, and I follow the convention of considering all teeth as molariforms (see González Ruiz et al., 2015). The cranial nomenclature follows Wible and Gaudin (2004). The nomenclature and analyses for osteoderms follow Goís et al. (2015) for pampatheres, Fernicola and Porpino (2012) for glyptodonts, and Krmpotic et al. (2009) for other armadillos. For both clades, the nomenclature used for the rest of the skeleton is specified for each species. No measurements were generated for the present study. Due to the large quantity of specimens, only the most complete or the diagnostic material was illustrated here. All specimens and associated information are available in Additional file 1: Table S1 (see Additional file 2: Appendix S1 for related references). Several specimens were documented digitally using X-ray micro-computed tomography (μCT). Specimens were scanned using a Nikon XT H 225 ST CT system. Each specimen concerned are mentioned in Additional file 1: Table S1.

### Geological setting

**Fig. 2:**
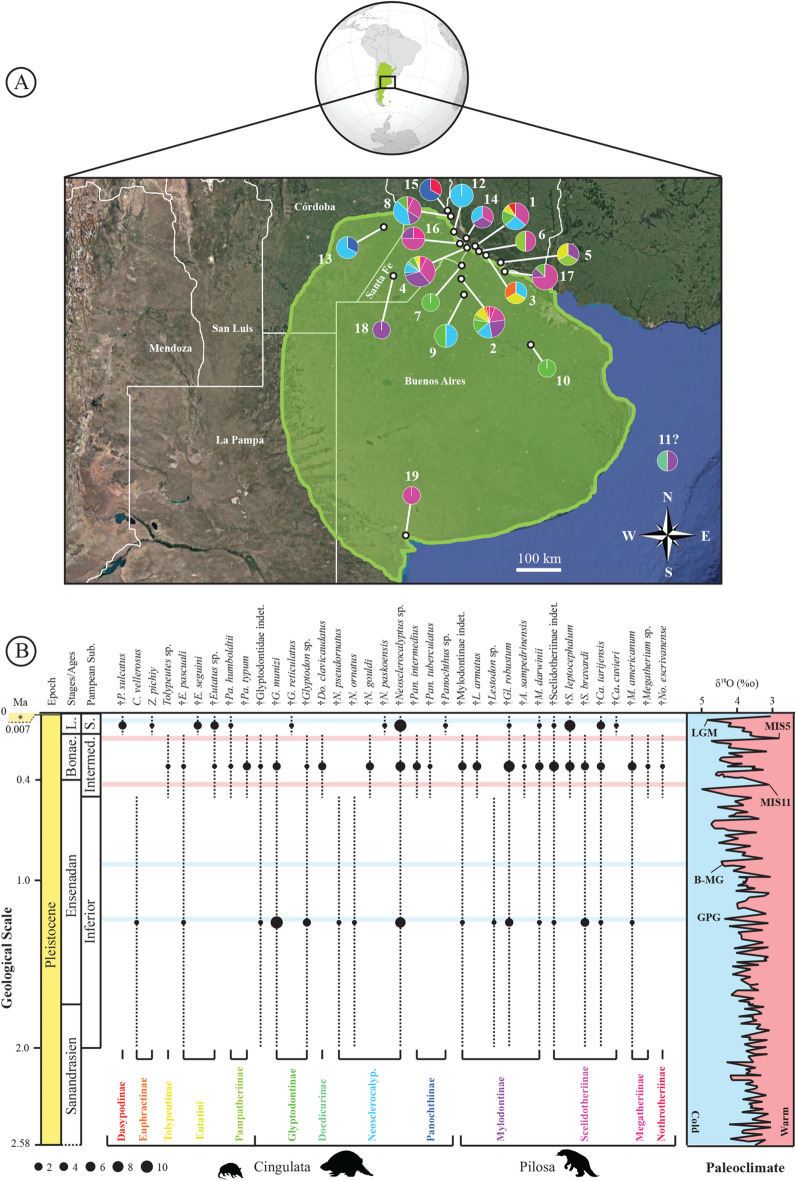
Localities and stratigraphies of xenarthrans from the Santiago Roth collection of the PIMUZ. **A** The map represents the humid Pampean Region (green) according to Prado et al. (2021). Each locality present in the collection is indicated by a number in agreement with Chichkoyan (2017) and Voglino (2020), and a diagram indicating the proportion of each xenarthra 'subfamily' found therein (see Additional file 1: Table S1 for more information and for locality names). The diagram size represents relative abundance, and the colors follow the stratigraphy. **B** The distribution shows the belonging of each species present in the collection after the taxonomic revision to the temporal subdivision of the geological scale according to Ages/Stages (see Cione & Tonni, 1995; Cione et al., 2015; Hansen, 2019; Prevosti & Forasiepi, 2018; Voglino, 2020). Distribution is associated with known Pleistocene paleoclimatic variation from the work of Zachos et al. (2001) and Solbeizon (2019). Circle size represents the abundance of each species for each subdivision. Symbol: *, Platan and Recent. Bonae., Bonaerian; B-MG, Matuyama/Brunhes Glaciation; GPG, Great Patagonian Glaciation; Intermed., Intermediate; L., Lujanian; LGM, Last Glacial Maximum; MIS, Marine Isotopic Stage; Neosclerocalyp., Neosclerocalyptinae; S., Superior; Sub., subdivision

All the specimens in the Roth collection at PIMUZ are from the Pampean Region, central east Argentina, more precisely from the humid pampa according to Prado et al. (2021), including Buenos Aires, Córdoba and Santa Fe (Fig. 2). However, the precise stratigraphic origin of the fossils is unknown on the basis of the information provided by Roth (1889). The sediments of the Pampean Region have been much discussed for a long time and were first considered as a single stratigraphical unit called the Pampean Formation (e.g., Bravard, 1857; Darwin, 1845; Orbigny, 1842) before undergoing multiple updates (e.g., Cione & Tonni, 1995; Cione et al., 2015; Hansen, 2019; Prado et al., 2021; Prevosti & Forasiepi, 2018; Voglino, 2020). Ameghino (1881) proposed splitting the Pampean Formation into three subdivisions, i.e., Lower Pampean, Upper Pampean, and Lacustrine Pampean based on lithology. These three subdivisions were used by Santiago Roth to assign each specimen to one of these units (Roth, 1889). Roth (1889) also renamed the subdivision of Ameghino (1881) as Inferior Pampean (earliest Pleistocene), Intermediate Pampean (Early and Middle Pleistocene), and Superior Pampean (Late Pleistocene). Nowadays, the Inferior, Intermediate, and Superior Pampean units are assigned to the late Sanandresian and a major part of the Ensenadan, the late Ensenadan and the Bonaerian, and the Lujanian, respectively (see Cione & Tonni, 1995; Cione et al., 2015; Hansen, 2019; Prado et al., 2021; Prevosti & Forasiepi, 2018; Voglino, 2020; and Fig. 2). No additional information can be addressed for the absolute age of each of the specimens. Here, I follow Santiago Roth's initial attribution and update with respect to the current chronostratigraphic scale. All the information corresponding to localities and relative ages is available in Additional file 1: Table S1 (see also Chichkoyan, 2017 and Voglino, 2020) and illustrated in Fig. 2.

## Results and discussions

### Taxonomic review of the Xenarthrans in the PIMUZ Santiago Roth collection

The material that Roth (1889) included in his 5th catalog corresponds to 284 fossil specimens. Among them, 150 specimens were xenarthrans, the vast majority of which correspond to the giants of the Pampean megafauna, i.e., glyptodonts and ground sloths. Among them, ten specimens were not found, and these are: a cranial fragment of a *Scelidotherium* Owen, 1839a of the Lujanian (No. 5 in the 5th catalog); two carapace fragments of a *Panochthus* Burmeister, 1866 of the late Ensenadan/Bonaerian (No. 69); teeth of an undetermined xenarthran (*Hoplophorus ornatus* Pouchet, 1866 according to Schulthess, 1920) supposedly from the Pleistocene of the Pampean region (No. 175); a cranial fragment of an undetermined ground sloth of the late Sanandresian/Ensenadan (No. 176); a tooth of a specimen identified as a *Mylodon* Owen, 1839a of the late Ensenadan/Bonaerian (No. 207); a fibula of an undertermined xenarthran of the late Sanandresian/Ensenadan (No. 219); a carapace fragment from a late Ensenadan/Bonaerian *Glyptodon* Owen, 1839b (No. 232); a fragment of a jaw and a cervical vertebra belonging to a specimen of *Hoplophorus* Lund, 1839 (= *Neosclerocalyptus* Paula Couto, 1857, see below) from the Lujanian (No. 233); a femur of a cingulate from the late Sanandresian/Ensenadan (No. 259); and a fragment of the carapace of a *Glyptodon* from the late Ensenadan/Bonaerian (No. 284). For the 140 remaining fossil specimens, completeness varies from isolated tooth fragments and osteoderms to nearly complete specimens. Only one specimen remains as an undetermined xenarthran (PIMUZ A/V 5694—No. 95), consisting of one incomplete tibia, one calcaneum and one-foot bone. The 139 remaining specimens are presented in this section and 114 are taxonomically reassigned. For each specimen, the initial assignation, original number from the 5th Roth catalog, locality, Stage/Age, and associated references are given in Additional file 1: Table S1.

Sysetematic Palaeontology

*Xenarthra* Cope, 1889

*Cingulata* Illiiger, 1811

*Dasypodidae* Gray, 1821

*Dasypodinae* Gray, 1821

*Propraopus* Ameghino, 1881

*Propraopus sulcatus* Lund, 1842

**Fig. 3:**
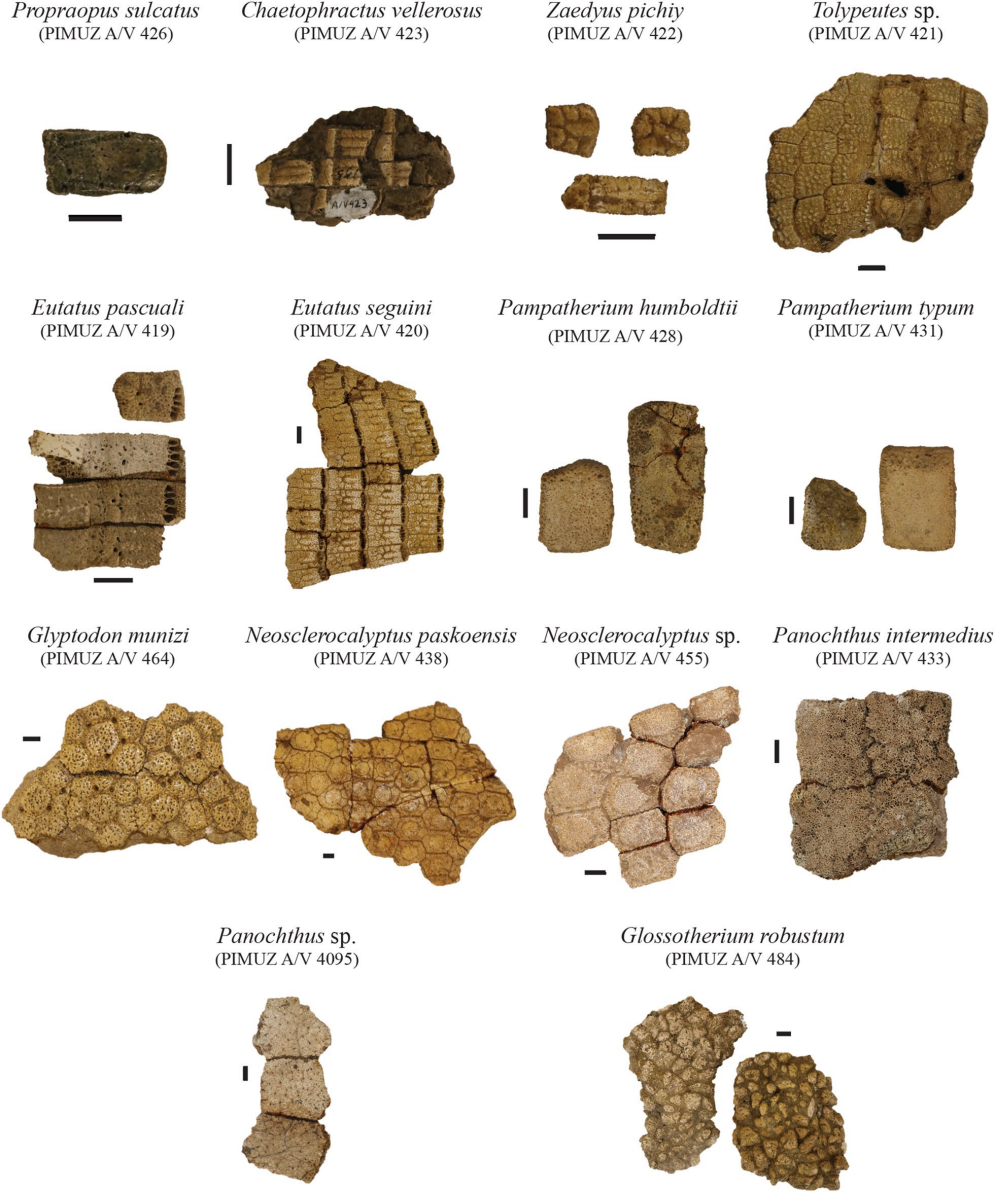
Plate of isolated or connected osteoderms for each species in the Roth collection at PIMUZ (from pelvic buckler, scapular buckler, or dorsal shield—see text). Almost all belong to the Cingulata, and the collection includes also osteoderms of Pilosa. Scale bar = 1 cm

**Referred material**: One isolated osteoderm: PIMUZ A/V 426 (Fig. 3); one isolated osteoderm: PIMUZ A/V 427.

**Comment**: Both specimens correspond to osteoderms from different parts of the dorsal shield. PIMUZ A/V 426 belongs to the mobile part. The unornamented part, also called the cranial portion (Krmpotic et al., 2009), is small, suggesting an anterior position of the osteoderm in the mobile shield (Schulthess, 1920). The specimen shows two main deep sulci bearing two foramina on one side and a foramen on the other, one of the synapomorphies of *Propraopus* (Winge, 1915). PIMUZ A/V 427 is cubic in shape with no apparent detail. It is likely that this osteoderm belongs to the peripheral part of the pelvic or scapular buckler, but the specimen does not allow further determination except that the size seems consistent with the assignment to the genus *Propraopus* (Winge, 1915). In agreement with the synonymy of *P. grandis* with *P. sulcatus* argued by Castro et al. (2013) and because the genus is monospecific including a diagnosis of *P. sulcatus* that fits with the two specimens in Roth's collection at PIMUZ, both specimens are therefore reassigned to *P. sulcatus*.

*Chlamyphoridae* Pocock, 1924

*Euphractinae* Winge, 1923

*Chaetophractus* Fitzinger, 1871

*Chaetophractus vellerosus* Gray, 1865

**Referred material**: Five subcomplete vertebrae in connection with three bony plates (= three bands) of which seven osteoderms of the movable bands from the dorsal shield are preserved: PIMUZ A/V 423 (Fig. 3).

**Comment**: PIMUZ A/V 423 was originally assigned to *Dasypus proximus* Lydekker, 1895 (Additional file 1: Table S1). In his work from 1887, Ameghino named the species *Proeuphractus proximus* Ameghino, 1887, later reassigned to *D. proximus* by Lydekker (1895). I could not find any taxonomic work mentioning this species. Yet Castro (2015) defined as valid species of the Dasypodini several genera and species, four of which occur in the Pleistocene Pampean region, one extinct, *P. sulcatus* (see above), and the others still extant *Dasypus hybridus* Desmarest, 1804, *Dasypus novemcinctus* Linnaeus, 1758, (or *Dasypus sabanicola* Mondolfi, 1968, see Feijó et al., 2019), and *Dasypus septemcinctus* Linnaeus, 1758, whose fossil record is unknown (Feijó, 2020). The vertebrae of PIMUZ A/V 423 are particularly broken and are not diagnostic. In contrast, the osteoderms are of small size and exhibit a large and smooth cranial portion, a well-marked transverse depression, and a caudal portion bearing two main parallel sulci (separating the central figure from the peripheral figures) with four foramina on each for the most complete osteoderm. None of the members of the Dasypodini mentioned above have parallel main sulci. These groove orientations often meet in the anteriormost part of the caudal portion in this Dasypodini (e.g., Castro, 2015). This feature is attributable to an Euphractinae, either belonging to the genus *Chaetophractus* or *Zaedyus* Ameghino, 1889, which is in agreement with their occurrence in the Ensenadan of Buenos Aires province (Soibelzon et al., 2010). Given the small size of the specimen, I favor a reattribution to *C. vellerosus* or *Z. pichiy* Desmarest, 1804 (Carlini et al., 2016; Superina & Abba, 2014). The only difference mentioned for a distinction of these two species on the osteoderms of the mobile bands is a less "sharp" shape in *C. vellerosus* (Carlini et al., 2016; Superina & Abba, 2014). As the specimen presents relatively flat and rectangular osteoderms, I favor its attribution to *C. vellerosus* but I note that this reattribution has to be taken with caution with regard to the little difference that exists with the species *Z. pichiy*. It is also to be noted that the recent phylogenetic analyses tend to discuss the relevance of differentiating these two species (e.g., Abba et al., 2015; Gibb et al., 2016). A point of interest brought by this specimen is the contribution of a partial axial skeleton in connection with the dorsal shield. The armadillo vertebral column has been studied mainly in *D. novemcinctus* for functional aspects (e.g., Gaudin & Biewener, 1992), or across extant armadillos in a study of the evolution of their axial skeleton (e.g., Galliari et al., 2010), but fossil specimens have rarely been the subject of comparative studies.

*Zaedyus* Ameghino, 1889

*Zaedyus pichiy* Desmarest, 1804

**Referred material**: Two osteoderms of the pelvic or scapular buckler, one osteoderm of the mobile bands of the dorsal shield, and one phalanx of the hind limb: PIMUZ A/V 422 (Fig. 3).

**Comment**: PIMUZ A/V 422 was initially assigned to *Dasypus patagonicus* Desmarest, 1819 (Additional file 1: Table S1), a species preliminarily defined by its small size. This species was later mentioned as a juvenile synonym of *Loricatus pichiy* Desmarest, 1804, occurring in the southern pampas of the province of Buenos Aires and placed in synonymy with *Z. pichiy* (Superina & Abba, 2014). These osteoderms bring me back to the same problem as for PIMUZ A/V 423 with doubt for the reattribution to *C. vellerosus* or to *Z. pichiy*. For PIMUZ A/V 422, the osteoderm of the mobile bands presents a sharper general shape and especially a stronger subdivision within the peripheral figures. In addition, the osteoderms of the pelvic and scapular bucklers show a general subquadratic shape and not rectangular as in *C. vellerosus*. Finally, the figures of these osteoderms exhibit a stronger convexity. Combined, all of these anatomical features tend to support the attribution of this specimen preferentially to *Z. pichiy* (Carlini et al., 2016; Superina & Abba, 2014).

*Tolypeutinae* Gray, 1865

*Tolypeutes* Illiger, 1811

*Tolypeutes* sp.

**Referred material**: Four plates of osteoderms from the dorsal shield belonging to the scapular and pelvic bucklers: PIMUZ A/V 421 (Fig. 3).

**Comment**: PIMUZ A/V 421 was originally assigned to *T. conurus* Geoffroy, 1847, a species placed in synonymy with the extant species *T. matacus* Desmarest, 1804 by Osgood (1919), referring to the reassessment of the mataco of Azara (1801) from Desmarest (1804). The osteoderms of the pelvic and scapular bucklers exhibit a central figure surrounded by peripheral figures composed of small tubercles. This configuration of the fixed osteoderms of the dorsal shield is a synapomorphy of *Tolypeutes* (see Soibelzon et al., 2010). The second valid species of the genus corresponds to *T. tricinctus* Linneaus, 1758, a species only present in the northeastern part of Brazil (Feijó et al., 2015), and thus excluded from the pampean region. There is also mention of an extinct species close to *T. matacus* named *T. pampaeus* Frenguelli, 1921, but the latter was synonymized with *T. matacus* due to lack of sufficient difference on the dorsal shield (Soibelzon et al., 2010). However, the distinction between the two valid species of the genus, i.e., *T. matacus* and *T. trincinctus*, in the diagnoses today corresponds only to the number of toes on the forefoot and the morphology of the scutes of the cephalic shield (see Magalhães et al., 2022). For PIMUZ A/V 421, only the exclusion of *T. tricinctus* from the Pampean region suggests an attribution of the specimen to *T. matacus*. Consequently, I favor in the present work an open taxonomy by reassigning PIMUZ A/V 421 to *Tolypeutes* sp.

*Eutatini* Bordas, 1933

*Eutatus* Gervais, 1867

*Eutatus pascuali* Krmpotic et al., 2009

**Fig. 4:**
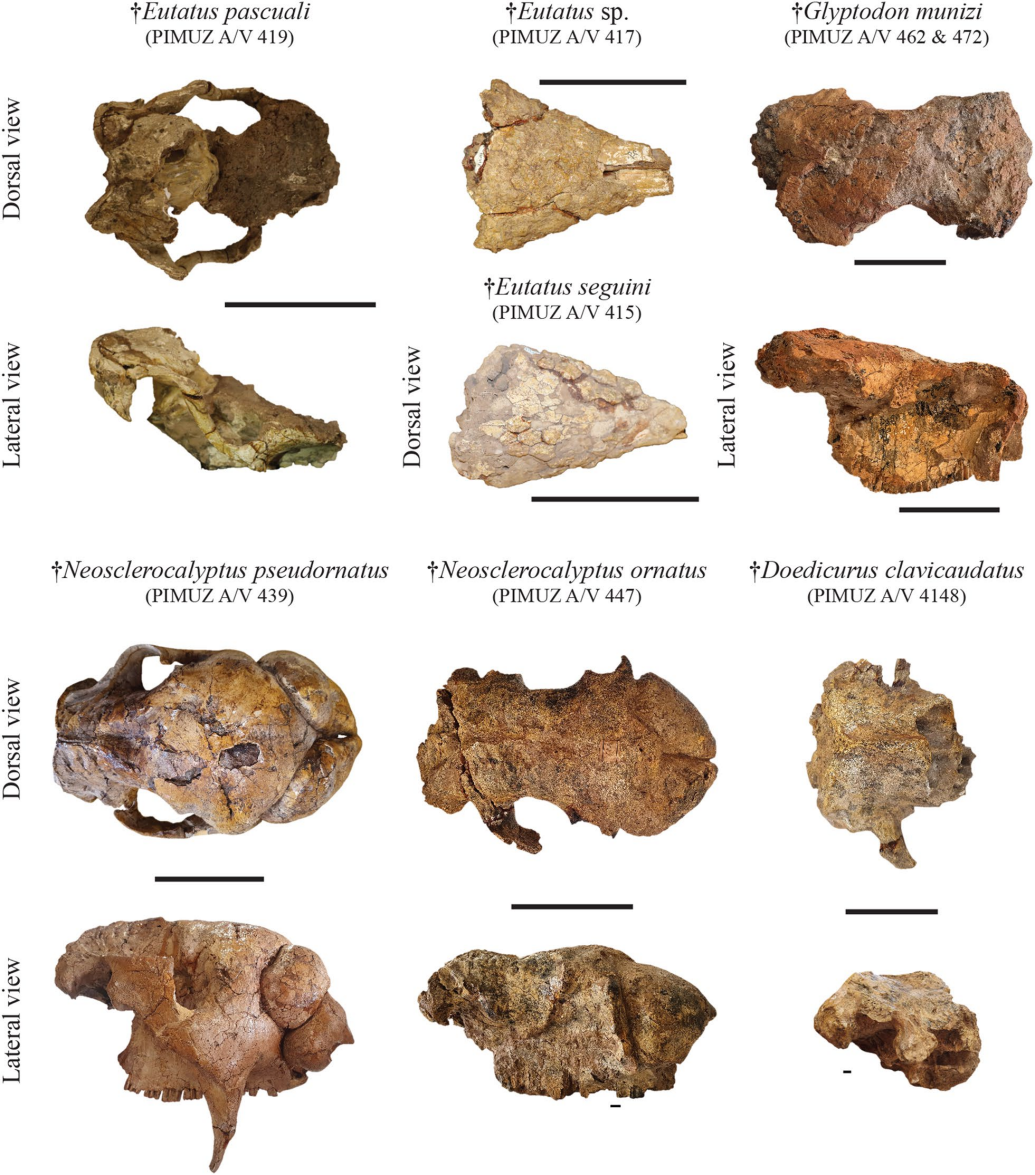
Plate of crania belonging to the Cingulata from the Roth collection at PIMUZ. The most complete remains illustrated here correspond to the Eutatini and glyptodonts. Scale bar = 10 cm

**Referred material**: Incomplete cranium bearing seven right molariforms and eight left molariforms, three undetermined bone fragments, two osteoderm from the pelvic buckler and nine osteoderms from the mobile bands of the dorsal shield: PIMUZ A/V 419 (Figs. 3, 4); one incomplete right femur: PIMUZ A/V 4094.

**Comment**: Members of the Eutatini are easily recognized by their molariforms composed of three distinct dentine layers and the presence of large hair foramina on the dorsal shield osteoderms (Krmpotic et al., 2009). Among genera of this clade, only the genus *Eutatus* is known from the Pleistocene of the Pampean region. Following a recent revision (Krmpotic & Scillato-Yané, 2007), the species *E. pascuali* is known to occur only in the Ensenadan while *E. seguini* Gervais, 1867 is typically Lujanian (Krmpotic et al., 2009). A new species from the Lujanian was also recently described, *E. crispianii* Brambilla & Ibarra, 2017, but this species shows foramina on the exposed surface larger than the hair foramina, which is not the case in our specimens. PIMUZ A/V 419 exhibits two pelvic shield osteoderms where the central figure is particularly large, with foramina on the exposed surface of a small size. The hair foramina are at a minimum number of five. These slight variations on these two osteoderms suggest an assignment to *E. pascuali* rather than *E. seguini*. In PIMUZ A/V 4094, there are no osteoderms available, which limits the determination. The relatively high position of the third trochanter on the femur is consistent with the assignment of this specimen to *E. pascuali* (Krmpotic et al., 2009). It is noteworthy that the cranium of PIMUZ A/V 419 shows a significant restored portion and there was probably an error in the reconstruction of this part of the cranium for this specimen.

*Eutatus seguini* Gervais, 1867

**Fig. 5:**
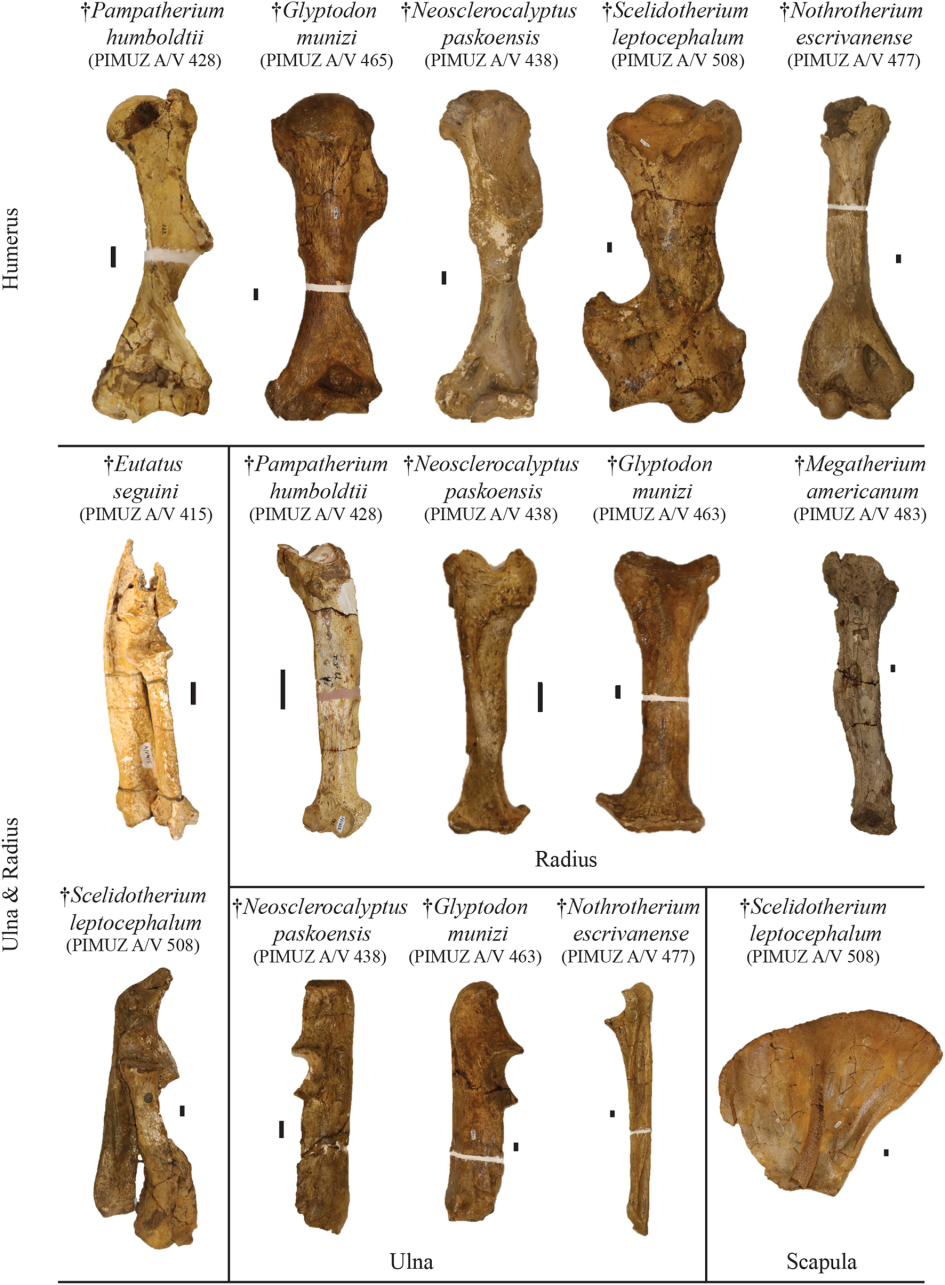
Plate of the most complete postcranial remains of the forelimb from the Roth collection at PIMUZ and a ground sloth scapula. Among the Cingulata, the best represented clades are large-sized armadillos (Eutatini, Pampatheriinae) and glyptodonts. In the ground sloths, only the mylodonts are not represented here. Scale bar = 1 cm

**Fig. 6:**
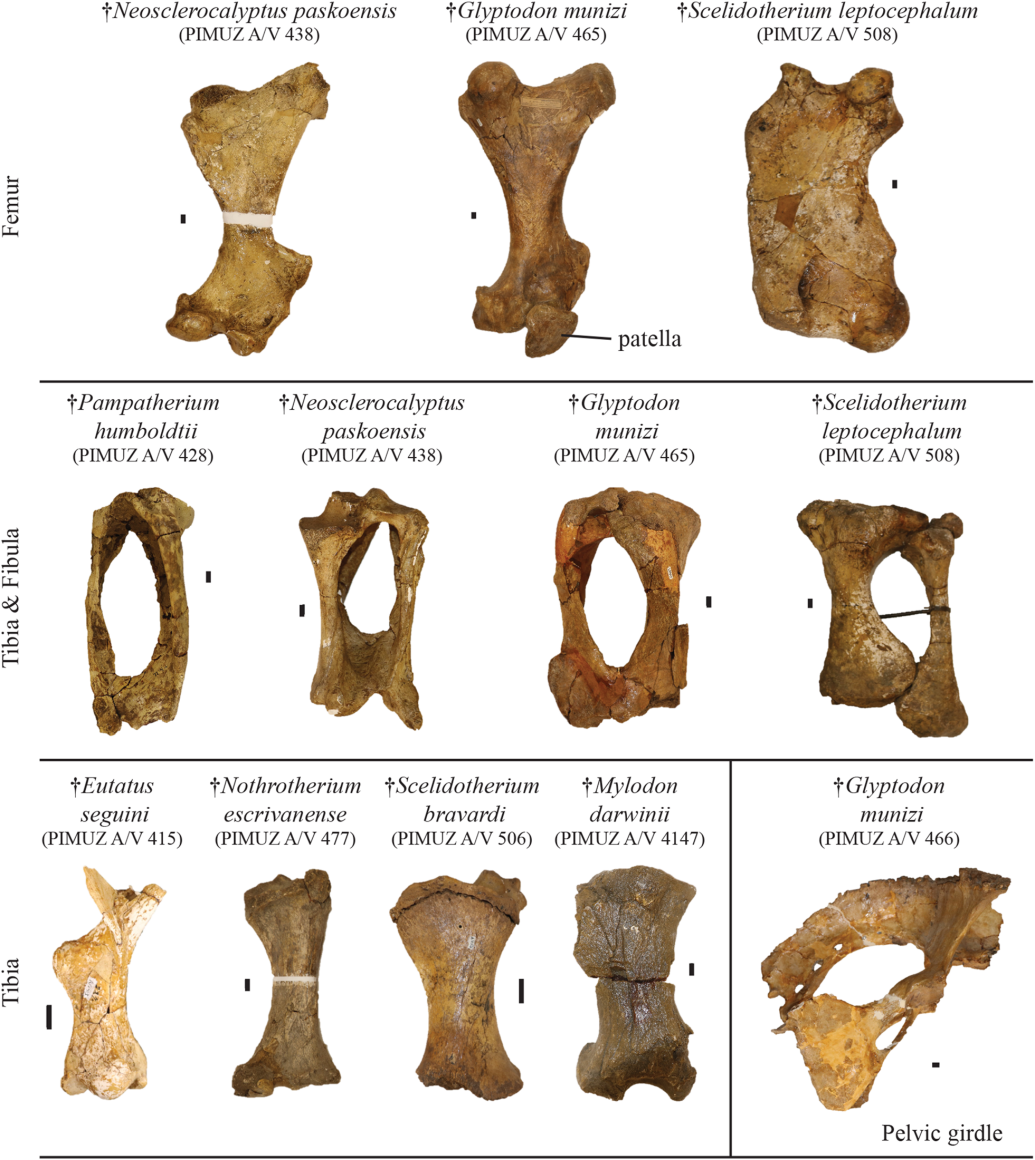
Plate of the most complete postcranial remains of the hindlimb from the Roth collection at PIMUZ and a glyptodont pelvic girdle. Among the Cingulata, the best represented clades are large-sized armadillos (Eutatini, Pampatheriinae) and glyptodonts. In the ground sloths, only the megatherines are not represented here. Scale bar = 1 cm

**Fig. 7:**
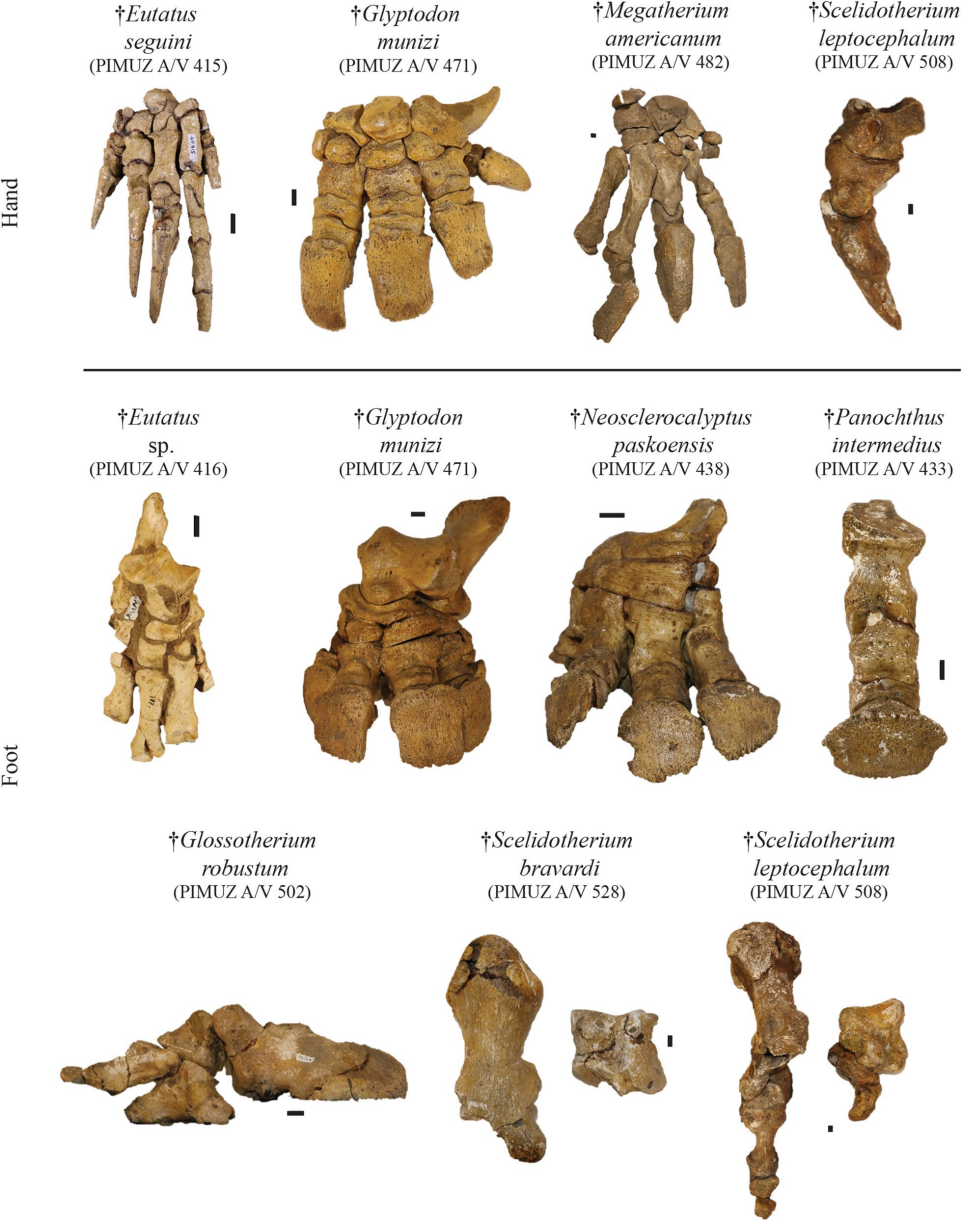
Plate of the most complete postcranial remains for the hand and the foot from the Roth collection at PIMUZ. For the hand of the cingulates, only an Eutatini and a glyptodont are presented and for the ground sloths, a megatherine and a scelidothere are illustrated to show associated remains. For the foot, the collection is richer with an Eutatini, several glyptodonts, a mylodont and a scelidothere represented here. Scale bar = 1 cm

**Referred material**: Anterior portion of the cranium bearing eight right molariforms and seven left molariforms, complete mandible bearing all teeth (broken), right humerus, right ulna, right radius, right hand, left fragmented ulna, left fragmented hand, and dorsal shield plates of osteoderms from the pelvic buckler: PIMUZ A/V 415 (Figs. 4, 5, 6, 7); one large plate of osteoderms from the pelvic buckler: PIMUZ A/V 420 (Fig. 3).

**Comment**: Compared to specimens assigned to *E. pascuali*, PIMUZ A/V 415 and PIMUZ A/V 420 exhibit osteoderms with relatively smaller central figures and larger exposed surface foramina. In particular, some osteoderms exhibit only four hair foramina, which support the initial attribution to *E. seguini* (Krmpotic et al., 2009). *E. seguini* was the initial proposed attribution for these specimens (Schulthess, 1920) and the few available distinctive elements tend to confirm these initial assignments.

*Eutatus* sp.

**Fig. 8:**
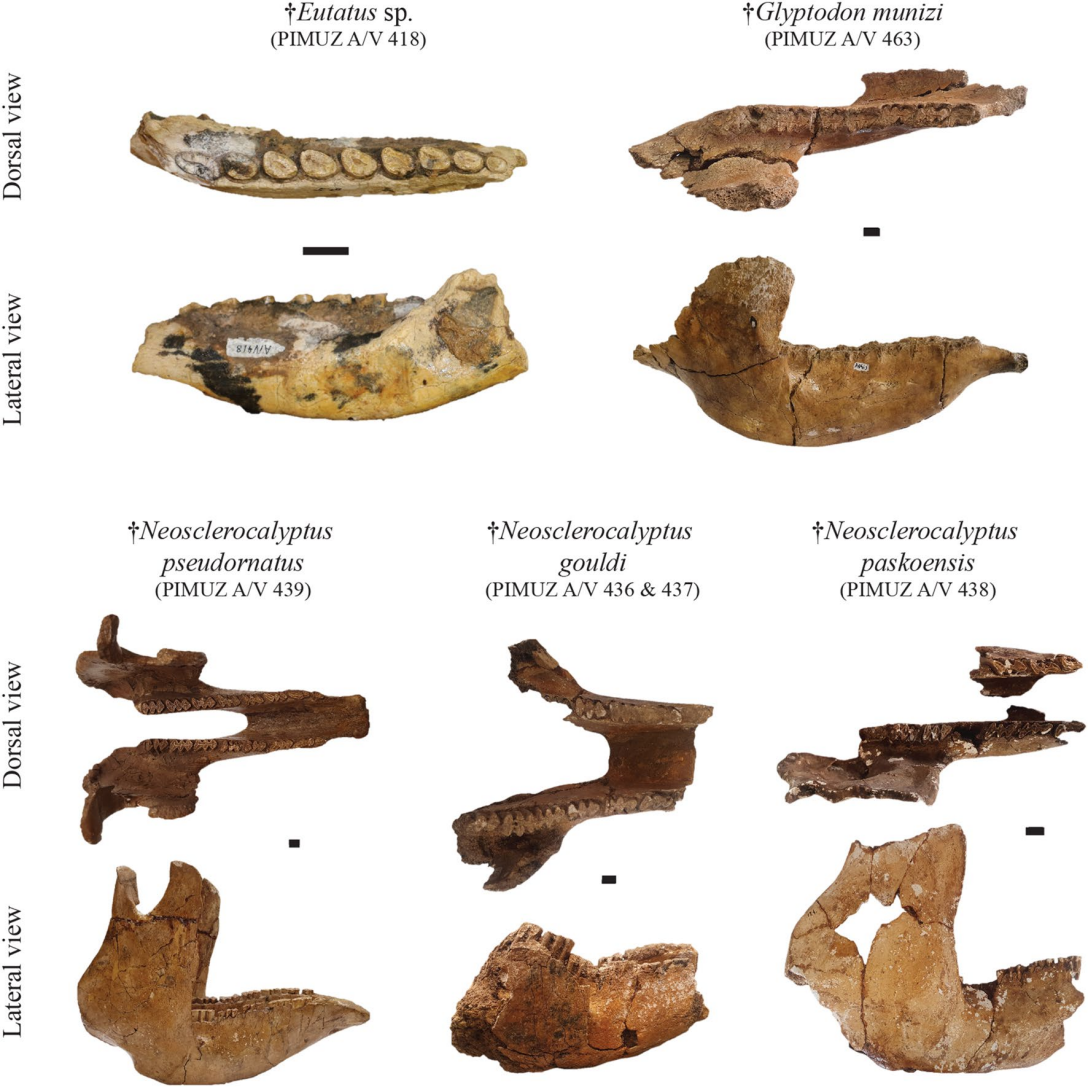
Plate of mandibles/hemimandibles belonging to the Cingulata from the Roth collection at PIMUZ. The most complete remains illustrated here correspond to the Eutatini and glyptodonts. Scale bar = 1 cm

**Referred material**: Incomplete right foot, one bone of the left foot, two fragmented plates of osteoderms from the dorsal shield: PIMUZ A/V 416 (Fig. 7); facial part of the cranium bearing part of the cephalic shield and one molariform on the left side of the jaw, and two altered plates of osteoderms from the dorsal shield: PIMUZ A/V 417 (Fig. 4); incomplete right mandible bearing nine molariforms: PIMUZ A/V 418 (Fig. 8); eight vertebrae in connection (six thoracic; two lumbar), two caudal vertebrae in connection, five isolated caudal vertebrae, and one fragmented long bone: PIMUZ A/V 4126.

**Comment**: In PIMUZ A/V 416 and PIMUZ A/V 417, the exposed surface of the osteoderms is not visible but the molariform present on the incomplete cranium allows to confirm the attribution to the genus *Eutatus*. The same remark is valid concerning the lower teeth of PIMUZ A/V 418. All specimens of this section do not allow a precise determination as bony features of the distal limbs, mandible, and vertebrae are not diagnostic at the species level (Brambilla & Ibarra, 2017; Krmpotic et al., 2009). For this reason, I prefer to invalidate the initial assignment to *E. seguini* (Schulthess, 1920) and instead propose an open taxonomy by assigning these specimens to *Eutatus* sp.

*Pampatheriinae* Paula Couto, 1954

*Pampatherium* Gervais & Ameghino, 1880

*Pampatherium humboldtii* Lund, 1839

**Referred material**: Right tibia, fibula, humerus, radius, 11 osteoderms of the scapular or the pelvic buckler and one osteoderm of the mobile bands of the dorsal shield: PIMUZ A/V 428 (Figs. 3, 5, 6); five osteoderms of the scapular or pelvic buckler: PIMUZ A/V 430.

**Comment**: All pampatheres in Roth collection at PIMUZ were originally assigned to the species *Chalmydotherium typum* Gervais & Ameghino, 1880. From the original description of the species *Pampatherium typum* Gervais & Ameghino, 1880, the use of the genus *Pampatherium* or *Chlamydotherium* Bronn, 1838, was much debated and remained unresolved for a long time. Since Ameghino (1891) synonymed *Chlamydotherium* with *Pampatherium*, several authors persisted in using *Chlamydotherium* without discussing the taxonomic validity of the genus (e.g., Winge, 1915). Nowadays, however, the validity of *Pampatherium* is no longer debated (Ferreira et al., 2018). From the Pampean region, according to Ferreira et al. (2018), four pampatheriine species have been described: *Holmesina paulacoutoi* Cartelle & Bohórquez, 1984, *Pa. humboldtii*, *Pa. typum* and *Tonnicinctus mirus* Góis et al., 2015. Identification of PIMUZ A/V 428 and PIMUZ A/V 430 represented by at least one fixed scapular or pelvic buckler osteoderm is based on Góis et al. (2015). These osteoderms differ in their general shape, rather pentagonal or hexagonal for the scapular buckler, and rather quadrangular for the pelvic buckler (Góis et al., 2015). *Pampatherium* differs from the other two genera by a narrower and less robust lateral and anterior margin, narrower anterior foramina, a less salient marginal elevation, and a shallower longitudinal depression (Góis et al., 2015). PIMUZ A/V 428 and PMIUZ A/V 430 are best assigned to *Pampatherium* as they show a well-defined non-smooth central figure as in *Pa. humboldtii* (Oliveira & Pereira, 2009). I therefore propose to reassign PIMUZ A/V 428 and PIMUZ A/V 430 to *Pa. humboldtii*. While the cranial and endocranial material is relatively well-studied for this species (e.g., Ferreira et al., 2018; Tambusso & Fariña, 2015a), the postcranial remains are poorly known. PIMUZ A/V 428 offers the opportunity to analyze several complete long bones of *Pa. humboldtii*. For instance, the addition of transitional size species between extant armadillos and glyptodonts, such as pampatheres, could be relevant to address issues concerning humeral shape and digging abilities in Cingulata (e.g., Milne et al., 2009).

*Pampatherium typum* Gervais & Ameghino, 1880

**Referred material**: Three osteoderms of the scapular or pelvic buckler: PIMUZ A/V 431 (Fig. 3); one osteoderm of the scapular buckler: PIMUZ A/V 432.

**Comment**: Regarding the genus attribution, the same remarks as for the previous section on *Pa. humboldtii* are applicable for PIMUZ A/V 431 and PIMUZ A/V 432—i.e., reassigned here to *Pampatherium*. Unlike PIMUZ A/V 428 and PIMUZ A/V 430, PIMUZ A/V 431 and PIMUZ A/V 432 exhibit a subtle delineation between the nearly smooth central figure and the lateral margins (Fig. 3). According to Oliveira and Pereira (2009), I thus assign these two specimens to *Pa. typum*.

*Glyptodontidae* Gray, 1869

Indet.

**Referred material**: Caudal tube fragment: PIMUZ A/V 473; part of the vertebral column: PIMUZ A/V 5154.

**Comment**: I refer here to Glyptodontidae specimen PIMUZ A/V 473 and PIMUZ A/V 5154, too altered to propose a ‘subfamily’ level assignment. PIMUZ A/V 5154 shows some osteoderms without any figures or tubercles associated with the vertebrae. This indicates an assignment to the Cingulata but the size of the column suggests a large specimen not suitable for large Eutatini and Pampatheriinae armadillos. I therefore propose to list this specimen as an undetermined glyptodont. For PIMUZ A/V 473, I encounter the same problem with the difference that it corresponds to a caudal tube which necessarily implies a belonging to one of the ‘subfamilies’ of glyptodonts except for Glyptodontinae, for which the caudal tube does not exist and leaves place to a caudal armor (Fernicola & Porpino, 2012).

*Glyptodontinae* Gray, 1869

*Glyptodon* Owen, 1839b

*Glyptodon munizi* Ameghino, 1881

**Fig. 9:**
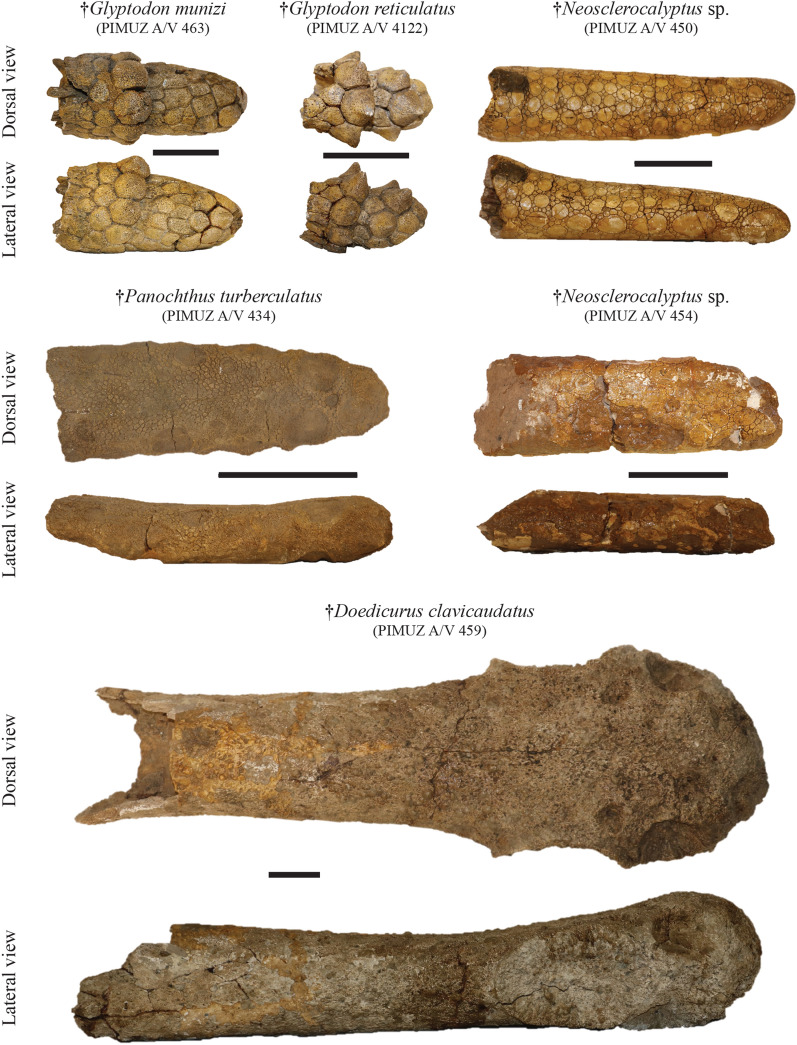
Plate of glyptodont caudal armors/tubes from the Roth collection at PIMUZ. Each Pleistocene glyptodont ‘subfamily’ is represented in the collection. Scale bar = 10 cm

**Referred material:** Almost complete skeleton: PIMUZ A/V 461 (Fig. 1); cranium and a large lateral border of the dorsal carapace: PIMUZ A/V 462 & 472 (Fig. 4); incomplete skeleton including mandible, five caudal vertebrae, distal caudal armor with associated vertebrae, one forelimb, isolated and associated foot bones, and caudal rings: PIMUZ A/V 463 (Figs. 5, 8, 9); sacral vertebra fragments, undetermined postcranial bones (pelvis?), 19 plates of osteoderms from the carapace, and 55 isolated or fragmented osteoderms: PIMUZ A/V 464 (Fig. 3); humerus, radius, right ulna and hand, left femur, right femur, tibia, and fibula, and plates of osteoderms from the carapace: PIMUZ A/V 465 (Figs. 5, 6); almost complete pelvic girdle: PIMUZ A/V 466 (Fig. 6); plates of osteoderms from the carapace: PIMUZ A/V 468; sacral vertebra fragment, caudal vertebra fragment, three fragments of the pelvic girdle, one plate of osteoderms from the carapace, and 21 isolated osteoderms: PIMUZ A/V 469; large plate of osteoderms from the carapace: PIMUZ A/V 470; three plates of osteoderms from the carapace, one isolated osteoderm, left hand, one foot: PIMUZ A/V 471 (Fig. 7); three plates of osteoderms from the carapace and two isolated osteoderms: PIMUZ A/V 4142; right mandible: PIMUZ A/V 4150.

**Comment**: Recent studies on the Glyptodontinae recognized only two genera for the Pleistocene, *Glyptotherium* Osborn, 1903, and *Glyptodon*. The first is not present in Argentina and occurs in South America in northern Venezuela and the eastern tip of Brazil (Zurita et al., 2018). I therefore focused on the genus *Glyptodon*, which was rich taxonomically in the past but taxonomic revisions have resulted in currently only three species being recognized (Cuadrelli et al., 2019, 2020). Only two species are known from the Pleistocene of the Pampean region: *G. munizi*, recorded only in the Ensenadan, and *G. reticulatus* Owen, 1845, recorded only in the Lujanian (Cuadrelli et al., 2019). Our taxonomic revision was therefore focused on the reassignment to one of these two species, considering also diagnostic features of the anatomy. PIMUZ A/V 461 and PIMUZ A/V 462 and 472 crania are assigned to *Glyptodon*. The former is complete and exhibits diagnostic features assignable to *G. munizi* such as a relatively circular orbital notch and a relatively shorter dorsoventral length of the zygomatic arches (Cuadrelli et al., 2019, 2020). However, PIMUZ A/V 461 shows descending processes of the zygomatic arches with a strong lateromedial extension accompanied by a slight medial curvature, characteristic of *G. reticulatus* (Cuadrelli et al., 2019). Both specimens show a postorbital constriction and a relatively strong fronto-parietal region index, morphologies that are more strongly marked in *G. munizi* (Cuadrelli et al., 2019, 2020). An assignment to *G. munizi* is also supported by a weak trilobation of Mf1, especially on the lingual margin, in both specimens (Cuadrelli et al., 2019, 2020). The attribution of PIMUZ A/V 461 to *G. munizi* is confirmed by the strong convexity of the dorsal carapace, while the part of the dorsal carapace preserved in PIMUZ A/V 462 and 472 corresponds to the caudal notch and shows a posterior elevation ending with strongly pronounced conical tubercles as in *G. munizi* (Cuadrelli et al., 2020). Independently of PIMUZ A/V 461, two specimens exhibit more or less well-preserved mandible remains. PIMUZ A/V 463 and PIMUZ A/V 4150 show mf1 and mf2 less lingually trilobate than in *G. reticulatus*, and especially their mf3 does not show furrow any in the anterolingual part of their first lobe, supporting an attribution to *G. munizi* (Cuadrelli et al., 2019). Apart from osteoderms, postcranial skeletal bones are only marginally diagnostic in differentiating the two species (Cuadrelli et al., 2019, 2020). Their major difference lies in the size, but this criterion does not seem robust, as it can be impacted by a strong intraspecific size variability, by ontogeny and by sexual dimorphism (see an example in *Glyptotherium*—Gillette & Ray, 1981; Gillette et al., 2016; Zurita et al., 2018). A recent study shows differences in pelvic girdle between *G. jatunkhirkhi* Cuadrelli et al., 2020, and *G. reticulatus* without addressing these comparisons to *G. munizi* (Cuadrelli et al., 2020). Based on the almost complete specimen, PIMUZ A/V 461, I can state that the pelvic girdle of PIMUZ A/V 466 has a much more dorsoventrally extended foramen obturator than in *G. reticulatus* but without the anteroposterior and dorsoventral extension of the ischial plate known in *G. jatunkhirkhi*. I hypothesize that this combination of foramen obturator and ischial plate is unique to *G. munizi*. For the rest of the specimens, our attention is focused on the osteoderms. For PIMUZ A/V 464, PIMUZ A/V 465, PIMUZ A/V 468, PIMUZ A/V 470, PIMUZ A/V 471, and PIMUZ A/V 4142, the osteoderms, or part of them, belong to the dorsal part of the carapace and present the typical rosette ornamentation known for *Glyptodon* (Cuadrelli et al., 2019; Fernicola & Porpino, 2012). In each of these osteoderms, a relatively flat central figure is surrounded by a line of peripheral figures always smaller than the central figure as in *G. munizi*. For PIMUZ A/V 469, there are only osteoderms in the lateroventral or posterior part of the carapace, accompanied by isolated conical tubercles of the caudal notch which are exactly similar to PIMUZ A/V 462 and 472 identified as belonging to *G. munizi* (see above). In conclusion, for all the specimens mentioned in this section, I propose a reattribution to *G. munizi* rather than to *G. reticulatus*. With 12 specimens belonging to *G. munizi*, the emblematic genus *Glyptodon* is the most represented cingulate species in the Roth collection at PIMUZ. The presence of an almost complete specimen underlines the strong potential for study of this species, but many other specimens present a relatively high degree of completeness for both cranial and postcranial remains. In recent years, investigations have focused particularly on endocranial structures (Le Verger et al., 2021; Tambusso & Fariña, 2015b; Tambusso et al., 2021). The cranial and endocranial anatomy of glyptodonts from the Roth collection at PIMUZ is discussed by Christen et al. (this volume).

*Glyptodon reticulatus* Owen, 1845

**Referred material**: Incomplete tail in five pieces and 20 isolated osteoderms from the tail: PIMUZ A/V 4122 (Fig. 9).

**Comment**: Specimen PIMUZ A/V 4122 from the Lujanian has a distal tip of the tail with thick osteoderms without a tip in contrast to the isolated osteoderms which all show a well-defined tip. These traits leave no doubt about the attribution to *Glyptodon,* but a more precise attribution is challenged by the close similarity of the tail between *G. munizi* and *G. reticulatus* (Cuadrelli et al., 2019, 2020). It is noteworthy that the distal tip of the tail does not present the robusticity known in *G. jatunkhirkhi*. According to Cuadrelli et al. (2019), the caudal armor of *G. reticulatus* is significantly smaller than the one of *G. munizi*, which is what I observe when I compare PIMUZ A/V 4122 with PIMUZ A/V 463, the latter being assigned to *G. munizi*. Because of this size difference, I favor an assignment of PIMUZ A/V 4122 to *G. reticulatus*, but it should be noted that this new assignment remains tenuous considering the reasons mentioned above. All so far described species of *Glyptodon* from the Pleistocene Pampean Region are represented in the PIMUZ collection; *G. munizi* is much more abundant than *G. reticulatus*. The difference in abundance could be due to a sampling bias both temporally and spatially, but this difference could also reveal a biological reality arising from potential competitiveness among the many glyptodont species present at the end of the Ensenadan (Fig. 2), or from environmental change induced by climate change occurring at the Bonaerian and the Lujanian (see below and Fig. 2), or a combination of these two factors.

*Glyptodon* sp.

**Referred material**: Nine isolated osteoderms: PIMUZ A/V 4096; mandible fragment: PIMUZ A/V 4097; nine undetermined postcranial bones: PIMUZ A/V 4155.

**Comment**: The three specimens in this section were originally assigned to the genus *Glyptodon* (Additional file 1: Table S1). Although the present study does not question this generic assignment, the specific attribution is questioned. PIMUZ A/V 4096 corresponds to several osteoderms on the lateral margins of the dorsal carapace, with a portion of the carapace that does not permit dissociation between *G. reticulatus* and *G. munizi*. PIMUZ A/V 4097 corresponds to a mandible fragment, but too incomplete to clearly assign the specimen to *G. munizi* or *G. reticulatus*. Finally, PIMUZ A/V 4155 shows several postcranial bones, a part of the skeleton that is only weakly diagnostic for both species. I therefore propose to assign PIMUZ A/V 4096, PIMUZ A/V 4097, and PIMUZ A/V 4155 to *Glyptodon* sp. in order to maintain an open taxonomy.

*Doedicurinae* Trouessart, 1897

*Doedicurus* Burmeister, 1874

*Doedicurus clavicaudatus* Gervais & Ameghino, 1880

**Referred material**: Almost complete caudal tube: PIMUZ A/V 459 (Fig. 9); complete neurocranium and anterior part of the left zygomatic arch: PIMUZ A/V 4148 (Fig. 4).

**Comment**: In his 5th catalog, Santiago Roth (1889) mentioned one specimen corresponding to a large glyptodont (No. 217 = PIMUZ A/V 459), later assigned to *Do. clavicaudatus* by Schulthess (1920), and one specimen corresponding to a "mysterious" glyptodont neurocranium (No. 215 = PIMUZ A/V 4148), both belonging to the late Ensenadan and the Bonaerian. During her revision, Schulthess (1920) attributed the neurocranium to *Doedicurus* sp. without providing details. Nowadays, the genus is considered monospecific and therefore contains only the species *Do. clavicaudatus* for which we know only well-identified remains in the Lujanian, although the diversity of the subfamily needs to be completely revised (see Núñez-Blasco et al., 2021). In the literature, the existence of *Doedicurus* in the Ensenadan was proposed by Ameghino (1889) on the basis of a revision of Burmeister's (1879) material, which led him to define the species *Doedicurus kokenianus* Ameghino, 1889. This new species was contested because the geographic provenances of the specimens used to define *Do. kokenianus* are imprecise. Lydekker (1895) synonymized *Do. kokenianus* with *Do. clavicaudatus*, a proposal rejected by Castellanos (1940). Several authors agree that a complete revision of the Pleistocene Doedicurinae is necessary (e.g., Núñez-Blasco et al., 2021; Soibelzon et al., 2010). Christen et al. have addressed the cranial anatomy of PIMUZ A/V 4148 in another study (this volume), but the determination of the specimen requires a deeper investigation within the Doedicurinae. Because of the current monospecific nature of *Doedicurus*, I preliminary consider the assignment to *Do. clavicaudatus* of PIMUZ A/V 459 (see Núñez-Blasco et al., 2021) and PIMUZ A/V 4148 (this volume). However, I encourage colleagues wishing to revisit the diversity of the Doedicurinae to consider these specimens from the Roth collection at PIMUZ either to extend the stratigraphic distribution of *Do. clavicaudatus* as proposed here, to revalidate the species *Do. kokenianus*, or to define a new species within the Doedicurinae as suggested by Roth (1889).

*Hoplophorinae* Huxley, 1864

*Neosclerocalyptinae* Porpino et al., 2010

*Neosclerocalyptus* Paula Couto, 1957

*Neosclerocalyptus pseudornatus* Ameghino, 1889

**Referred material**: Complete skull, cervical vertebrae, 24 fragments of vertebrae and other undetermined bones: PIMUZ A/V 439 (Figs. 4, 8).

**Comment**: The diversity of the Hoplophorinae has been long debated and the validity of many genera has been questioned (e.g., Fernicola, 2008; Paula Couto, 1957; Zurita et al., 2007). A large part of the diversity originally attributed to *Hoplophorus* is now accepted to belong to the Neosclerocalyptinae on the basis of strong differences in cranium and carapace morphology (Porpino et al., 2010), as it is the case for many specimens from the Pampean region (Zurita et al., 2009b). Currently, only one species of *Hoplophorus* is accepted as valid from the Pleistocene of Brazil (Porpino et al., 2010). The set of specimens from the Roth collection attributed to *Hoplophorus* must therefore be revised. The Neosclerocalyptinae are small glyptodonts (~300-600 kg) compared to other ‘subfamilies’ from the Pleistocene (Vizcaíno et al., 2011). This ‘subfamily’ is the most abundant in the fossil record (Carlini et al., 2008; Zurita et al., 2009a, b), as also it is in the Roth collection at PIMUZ. Species belonging to *Neosclerocalyptus* exhibit several singular cranial features associated with strong ossification of the nasal cartilage (Zurita et al., 2011). The variations in snout shape due to this atypical ossification have been used to distinguish the different species belonging to *Neosclerocalyptus* (Zurita et al., 2011). PIMUZ A/V 439 exhibits a snout consisting of two bulbs, derived from ossification of the nasal cartilage, with a low narial opening directed anteroventrally. The organization into two bulbs arranged one above the other is the main autapomorphy of the species *N. pseudornatus* and would mark a weaker pneumatization of the sinuses compared to other Pleistocene species for which the narial opening is much larger (Zurita et al., 2011). I also note that a nearly identical specimen was illustrated in the work of Zurita et al., (2011; Fig. 2A–D – no collection number specified) in support of the identification of PIMUZ A/V 439 to *N. pseudornatus*. Because of its exceptional preservation, the skull of PIMUZ A/V 439 could be subject for the study of Neosclerocalyptinae. In the literature, the ‘subfamily’ has been hypothesized to present a specific diversity resulting from anagenesis induced by a potential correlation between the development of nasal cartilage ossification and the increase of aridity during the cooling phases of the Pleistocene (Zurita et al., 2011). This hypothesis was challenged by an investigation of the paranasal sinuses and the nasal cavity of several glyptodonts (Fernicola et al., 2012), but this latter investigation was focused on the most derived *Neosclerocalyptus paskoensis* Zurita, 2002, which has also the most extensive pneumatization. A study including the other *Neosclerocalyptus* species should be conducted to test this hypothesis. The exploration of the endocranial cavities of PIMUZ A/V 439 may provide further insight into this hypothesis and, therefore, for illuminating the evolution of the clade.

*Neosclerocalyptus ornatus* Owen, 1845

**Referred material**: Cranium, 13 plates of osteoderms from dorsal carapace, and 61 isolated or fragmented osteoderms: PIMUZ A/V 447 (Fig. 4).

**Comment**: The cranium of PIMUZ A/V 447 exhibits more developed fronto-nasal sinuses than *N. pseudornatus*, with a ventrally oriented 'funnel'-shape, showing a V-shape cleft separating the frontal from the maxilla. The narial opening is larger than in *N. pseudornatus* and the pneumatization is more strongly developed but without reaching the stages of *Neosclerocalyptus gouldi* Zurita et al., 2008, and *N. paskoensis* (Zurita et al., 2011). I therefore propose a reattribution of PIMUZ A/V 447 to *N. ornatus*. The presence of the cranium PIMUZ A/V 447 will also allow comparison of the endocranial cavities of the two oldest *Neosclerocalyptus* species from the Pampean Region (see above).

*Neosclerocalyptus gouldi* Zurita, Carlini & Scillato-Yané, 2008

**Referred material**: Incomplete part of the neurocranium (including ear ossicles), two fragments of mandible, right tibia and fibula (only distal parts preserved), isolated foot bones, fragment of cephalic shield, seven plates of osteoderms from the carapace: PIMUZ A/V 436 and 437 (Fig. 8); two cephalic shields: PIMUZ A/V 458.

**Comment**: PIMUZ A/V 436 and 437 exhibits a strong trilobation of mf2 and PIMUZ A/V 458 shows pronounced lateral curvatures of the cephalic shield, two anatomical features that support a preferential assignment to *N. gouldi* among *Neosclerocalyptus* species (Zurita et al., 2008). The Roth collection at PIMUZ is of interest by the presence of specimen PIMUZ A/V 436 and 437 for which the ear bones are preserved. Schulthess (1920) stated that the specimen probably corresponds to a young individual, but I am unable to find arguments to confirm this statement except for the relatively small size, but remaining close to the size range of adult specimens (Zurita et al., 2008). Indeed, an analysis of ontogenetic variation would be useful for many species of glyptodonts from southern South America.

*Neosclerocalyptus paskoensis* Zurita, 2002

**Referred material:** Right mandible, incomplete left mandible, several tooth fragments, cervical vertebra, seven vertebrae, humerus, right and left radius, ulna, and foot, 32 postcranial fragments, six large plates of osteoderms from the carapace, four annular rings, 59 isolated or fragmented osteoderms: PIMUZ A/V 438 (Figs. 3, 5, 6, 7, 8).

**Comment**: Although PIMUZ A/V 438 corresponds to an almost complete skeleton, the absence of a complete cranium or carapace makes its determination difficult. This specimen still has an incomplete mandible and, of all *Neosclerocalyptus* species, *N. paskoensis* has the most gracile mandible (Zurita et al., 2008), an anatomical feature I observe in PIMUZ A/V 438 compared to other specimens in the Roth collection at PIMUZ. I therefore favor a reassignment of specimen PIMUZ A/V 438 to the species *N. paskoensis*. However, to confirm this description, a complete review of the genus *Neosclerocalyptus* is necessary, together with an improvement of their diagnoses.

*Neosclerocalyptus* sp.

**Referred material:** One tooth: PIMUZ A/V 440; one tooth: PIMUZ A/V 441; incomplete hemimandible and one osteoderm: PIMUZ A/V 442; one tooth: PIMUZ A/V 443; one phalanx: PIMUZ A/V 445; one phalanx: PIMUZ A/V 446; one plate of osteoderms from the carapace: PIMUZ A/V 448; one isolated osteoderm: PIMUZ A/V 449; caudal tube, two annular rings, 12 plates of osteoderms from the carapace, 47 isolated or fragmented osteoderms: PIMUZ A/V 450 (Fig. 9); large plate of osteoderms from the carapace: PIMUZ A/V 451; fragmented caudal tube: PIMUZ A/V 452; six plates of osteoderms from the carapace and five isolated osteoderms: PIMUZ A/V 453; fragmented caudal tube: PIMUZ A/V 454 (Fig. 9); one plate of osteoderms from the carapace: PIMUZ A/V 455 (Fig. 3); one plate of osteoderms from the carapace: PIMUZ A/V 456; one isolated osteoderm: PIMUZ A/V 457; navicular: PIMUZ A/V 474; navicular: PIMUZ A/V 475; three plates of osteoderms from the carapace and 37 isolated osteoderms: PIMUZ A/V 476; 13 plates of osteoderms from the carapace: PIMUZ A/V 4124; 22 plates of osteoderms from the carapace and 150 isolated osteoderms: PIMUZ A/V 4135; two plates of osteoderms from the carapace: PIMUZ A/V 4141; 13 plates of osteoderms from the carapace: PIMUZ A/V 4251.

**Comment**: For all specimens of this section, there is no associated complete cranium or carapace. As the diagnoses of each species of *Neosclerocalyptus* focus mainly on cranial characters or overall carapace characters (Zurita et al., 2009b), it is impossible to distinguish some species on the basis of fragmentary material. This is particularly the case for the two oldest species from the Ensenadan, *N. pseudornatus* and *N. ornatus*. On the postcranial material, I can add that the differences between *N. gouldi*, the species presents at the end of the Ensenadan and the Bonaerian, and the older species, *N. pseudornatus* and *N. ornatus*, are particularly weak (Zurita et al., 2008). However, identification at the genus level is consistent and can be supported by the osteoderm pattern. The set of osteoderms in the remaining specimens follows the genus diagnosis, with dorsal carapace osteoderms exhibiting relatively ‘primitive’ ornamentation close to Propalaehoplophorinae (Zurita et al., 2009b), i.e., the osteoderms are thin and large with a flat central figure always wider than the peripheral figures of the surrounding line, the demarcation between the figures are well delineated by deep sulci (Zurita et al., 2009b). Quantification of the proportions of central and peripheral figures might be useful in the search for diagnostic elements to recognize *Neosclerocalyptus* species regardless of the cranial remains and the general profile of the dorsal carapace. Specimen PIMUZ A/V 450 also consists of a well-preserved caudal tube. The latter shows six oval figures on the lateral margins that progressively increase in size toward the most distal part. The apex of the caudal tube ends in large terminal dorsolateral figures. This general osteoderm organization of the caudal tube is a characteristic of the genus *Neosclerocalyptus* (Zurita et al., 2009b), but it does not carry enough differences to distinguish the species. On this basis, also PIMUZ A/V 452 and PIMUZ A/V 454 can be assigned to *Neosclerocalyptus*. Since the taxonomical revision implies a reassignment at the level of the genus from the initial assignments (Schulthess, 1920; see Additional file 1: Table S1), and because there are no major differences with the osteoderms of the well-identified specimens (i.e., PIMUZ A/V 447), I propose to reassign all specimens from this section including osteoderms or caudal tube to *Neosclerocalyptus* sp., pending potential new postcranial diagnostic elements to differentiate the species of *Neosclerocalyptus*. PIMUZ A/V 440, PIMUZ A/V 441, PIMUZ A/V 443, PIMUZ A/V 445, PIMUZ A/V 446, PIMUZ A/V 474, and PIMUZ A/V 475 correspond to either an isolated tooth or an isolated postcranial bone, preventing a clear taxonomic identification. These seven specimens were initially assigned to the species *Hoplophorus ornatus*, with the exception of PIMUZ A/V 474 which was assigned to *Hoplophorus* sp. (Schulthess, 1920). However, *Hoplophorus ornatus* is no longer a valid species and was defined as a synonym of *N. ornatus* (see Porpino et al., 2010). Therefore, I propose only to follow synonimization at the generic level and maintain an open taxonomy by assigning these seven specimens to *Neosclerocalyptus* sp. It is noteworthy that all specimens seem to be adults with the exception of PIMUZ A/V 442, which appears to be a young or a newborn specimen. The incomplete hemimandible bears three incompletely erupted teeth, including mf4, the first tooth to erupt in the lower dentition in glyptodont (González Ruiz et al., 2020). The teeth have a conical appearance with a less marked trilobulation than in the adult stage. The shape of the teeth at such a young ontogenetic stage is reminiscent of the recently published juvenile specimen of an Early Miocene glyptodont (González Ruiz et al., 2020) and is of particular interest for the study of glyptodont dentition development and the evolution of hypso/hypselodonty within the clade.

*Panochthinae* Burmeister, 1866

*Panochthus* Burmeister, 1866

*Panochthus intermedius* Lydekker, 1895

**Referred material:** caudal vertebrae, left incomplete hand, left incomplete hind limb, right incomplete foot, 16 sesamoid bones, 34 small plates of osteoderms from dorsal carapace, and 37 isolated osteoderms: PIMUZ A/V 433 (Figs. 3, 7); 15 plates of osteoderms from dorsal carapace and 14 isolated osteoderms: PIMUZ A/V 435.

**Comment**: A recent study produced the comparative description of *Panochthus* species from the Pampean Region from the Ensenadan (Zamorano et al., 2021). The authors support the presence of just three species of *Panochthus* in this region at this period: *Pan. intermedius*, *Panochthus subintermedius* Castellanos, 1937, and *Panochthus* cf. *Pan. subintermedius* (Zamorano, 2012). While the shape and size of the fingers and toes of PIMUZ A/V 433 are consistent with assignment to the genus (see Schulthess, 1920), the main argument for the determination is supported by the ornamentation of the multiple osteoderms corresponding to numerous polygonal tubercles equal in size on a single osteoderm (Zamorano et al., 2014a, 2021). This feature is unique to certain regions of the dorsal carapace of *Pan. intermedius* and *Pan. subintermedius*, mentioned above, but also to the Lujanian species, *Pan. tuberculatus* and *Pan. frenzelianus*. Among the species from Ensenadan, a distinction is difficult with isolated osteoderms because it is made according to the region of the carapace where central figures are found, the antero- and posterodorsal regions for *Pan. intermedius* and only the last row of the posterior margin of the carapace for *Pan. subintermedius* (Zamorano et al., 2021). In PIMUZ A/V 433 and PIMUZ A/V 435, many osteoderms show no central figure, which indicates other regions of the dorsal carapace with more hexagonal shapes for the more dorsal part, and more quadrangular shapes for the more lateral part (Zamorano et al., 2021). However, for both specimens some osteoderms exhibit a central figure and none of these seem to belong to the last row of the posterior margin of the carapace, indicating a clear assignment of PIMUZ A/V 433 and PIMUZ A/V 435 to *Pan. intermedius* (Zamorano et al., 2021). Because *Pan. intermedius* is significantly larger than *Pan. subintermedius*, *Pan. tuberculatus*, and *Pan. frenzelianus* (e.g., Zamorano et al., 2014a), another possible distinction is directly dependent on relative osteoderm size. In addition, the tubercles of each osteoderm type, hexagonal or quadrangular, of PIMUZ A/V 433 and PIMUZ A/V 435 are particularly large and appear to be closer to *Pan. intermedius* than to *Pan. subintermedius* (Cruz et al., 2011; Zamorano et al., 2014a, b, 2021). Accordingly, I propose to reassign these specimens to *Pan. intermedius*.

*Panochthus tuberculatus* Owen, 1845

**Referred material:** Caudal tube: PIMUZ A/V 434 (Fig. 9).

**Comment**: PIMUZ A/V 434 is from the late Ensenadan/Bonaerian for which only three species are known (see previous section). The main elements of identification for the caudal tube lie in the comparison of the distribution of figures of the distal part of the caudal tube in dorsal view. As mentioned by Brambilla et al. (2020) and Zamorano & Fariña (2022), only *Pan. tuberculatus* exhibits symmetrical main dorsal figures between which there is distally a central main figure followed by asymmetrical apical figures towards the tip of the caudal tube. This distribution is found in PIMUZ A/V 434, supporting the initial attribution to *Pan. tuberculatus* (Schulthess, 1920; see Additional file 1: Table S1). While the identification is clear, the age of the specimen implies an older origin of *Pan. tuberculatus* which is not recognized today as present in late Ensenadan/Bonaerian. With the presence of PIMUZ A/V 434, the Roth collection at PIMUZ has at least one tail from each major glyptodont ‘subfamily’.

*Panochthus* sp.

**Referred material:** plates of osteoderms from dorsal carapace: PIMUZ A/V 4095 (Fig. 3).

**Comment**: The osteoderms of PIMUZ A/V 4095 are similar to the previously treated specimens but are from the Lujanian, a period for which the presence of *Pan. tuberculatus* and *Pan. frenzelianus* is recognized (Zamorano et al., 2021). While *Pan. tuberculatus* is a well-known species, the validity of *Pan. frenzelianus* is debated (Zamorano et al., 2021). However, although the osteoderms exhibit a characteristic pattern of the genus *Panochthus* (see above), there is no reason to propose a more precise determination on the basis of this material. I therefore favor a more open taxonomy for this specimen by assigning PIMUZ A/V 4095 to *Panochthus* sp.

*Pilosa* Flower, 1883

*Folivora* Delsuc et al., 2001

*Mylodontidae* Gill, 1872

*Mylodontinae* Gill, 1872

Indet.

**Referred material:** First molariform: PIMUZ A/V 489; upper molariform: PIMUZ A/V 490; incomplete pelvis: PIMUZ A/V 500; tibia: PIMUZ A/V 4099; fragments of the mandible: PIMUZ A/V 4101.

**Comment**: The specimen PIMUZ A/V 4099 is similar in all respects to the specimen PIMUZ A/V 4147 attributed to *Mylodon darwinii* Owen, 1839a (see below) with the difference that the epiphyses are not sufficiently preserved to document the articular facets. This alteration of the epiphyses, especially the distal part as it carries the diagnostic elements, limits our identification. The mandibular fragments are particularly uninformative, and the specimen shows an unnatural organization of the teeth. I suspect that different pieces were incorrectly assembled. The one relatively complete tooth could be an mf1 of *Mylodon* Owen, 1839a (McAfee, 2009) but I cannot confirm this statement. PIMUZ A/V 489 and PIMUZ A/V 490 correspond to isolated teeth without any particularity except for an oval shape longer than wide, suggesting an attribution to mf1, which could belong to different species of mylodonts. Finally, the specimen PIMUZ A/V 500 is a pelvic girdle too fragmentary to suggest a clear determination (but see Cartelle et al., 2019 for an example of mylodont pelvis girdle). As a precaution, I prefer to propose uncertainty at the ‘subfamilial’ rank to limit questionable interpretations for the specimens mentioned in this section.

*Lestodon* Gervais, 1855

*Lestodon armatus* Gervais, 1855

**Fig. 10:**
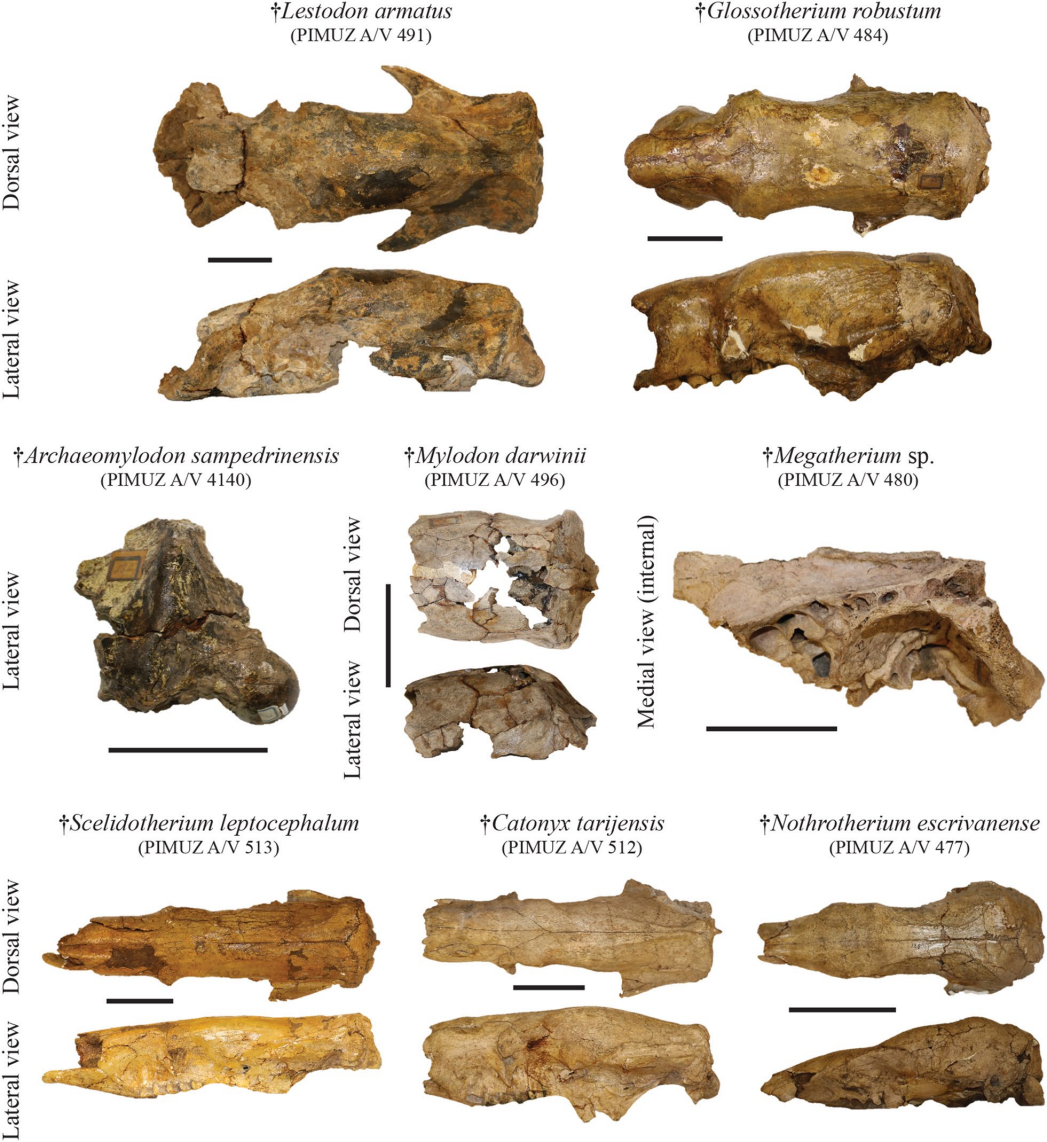
Plate of crania belonging to the Pilosa from the Roth collection at PIMUZ. Each ‘subfamily’ presents cranial remains among which the most complete belong to the Mylodontinae, the Scelidotheriinae and the Nothrotheriinae. Scale bar = 10 cm

**Fig. 11:**
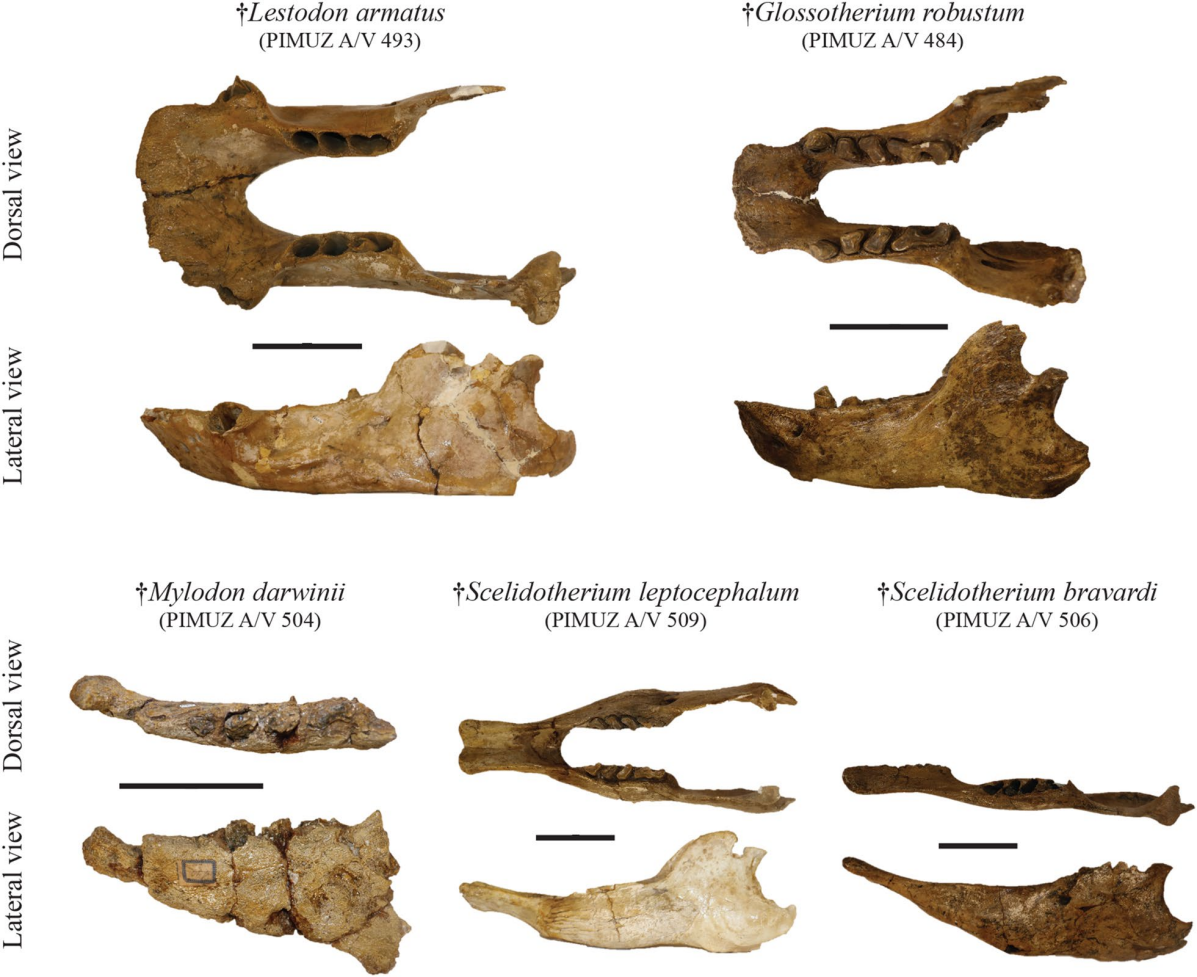
Plate of mandibles/hemimandibles belonging to the Pilosa from the Roth collection at PIMUZ. The most complete remains illustrated here correspond to mylodonts and scelidotheres. Scale bar = 10 cm

**Referred material:** Cranium: PIMUZ A/V 491 (Fig. 10); right mandible bearing four teeth, upper left jaw bearing four teeth, one tibia fragment, one phalanx, one rib: PIMUZ A/V 492; ribs and one mandible: PIMUZ A/V 493 (Fig. 11); one caniniform: PIMUZ A/V 494.

**Comment**: The genus *Lestodon* was exclusively diagnosed on the basis of cranial and dental characters with a U- or V-shaped nasal-frontal suture, a prezygomatic constriction, a snout roof consisting only of the nasal compared to other ground sloths (Bargo et al., 2006a), and a strong diastema separating the large triangular caniniform from the molariforms (Esteban, 1996; Pascual et al., 1966). Long considered highly diverse, *Lestodon* is now recognized as monospecific by containing the single species *Lestodon armatus* (Czerwonogora & Fariña, 2013). PIMUZ A/V 491 corresponds to an almost complete cranium possessing all the diagnostic characteristics of the genus. The strongly pronounced diastema is also found between the cf1 and the mf1 in the mandibles of PIMUZ A/V 492 and PIMUZ A/V 493. Although the hemimandible of PIMUZ A/V 492 belongs to a particularly young specimen, a lateral widening at the front of the caniniform forms a large shelf clearly visible in the adult specimen PIMUZ A/V 493. The latter has only triangular caniniforms while the three molariform ones are absent. The alveolar cavity of mf3 is bilobate with an anterior lobe larger than the posterior; this dental shape is present in the last molariform in *Lestodon* (e.g., Varela et al., 2022). PIMUZ A/V 494 exhibits only the tip of a triangular caniniform that appears triangular in cross-section. Although the tooth is not complete, its size suggests it belonged to a small specimen highlighting the presence of a second juvenile of *L. armatus* in the Roth collection at PIMUZ. For each of the mentioned specimens, a reassignment to the genus *Lestodon* seems to correspond, even for the most fragmentary remains. I therefore propose a reassignment of these specimens to *L. armatus*. For this species, the major scientific interest lies in the completeness of the skull PIMUZ A/V 491 allowing analyses of the cranial shape but also to explore the endocranial structures, which are still unknown. The presence of several well-preserved vertebrae also offers the possibility to functional studies on the axial skeleton of ground sloths. However, the most relevant specimens are the juveniles in the Roth collection at PIMUZ because the morphological variation resulting from ontogeny is still poorly known for *Lestodon*.

*Lestodon* sp.

**Referred material:** One incomplete femur and ten vertebrae: PIMUZ A/V 503.

**Comment**: The taxonomic revision is difficult for PIMUZ A/V 503 because the specimen is represented only by vertebrae and an incomplete and fragmentary femur. While a recent study has provided new diagnostic evidence for postcranial material of *Lestodon* (Vargas-Peixoto et al., 2021), the vertebrae do not allow for a genus identification and the femur is too fragmentary (Vargas-Peixoto et al., 2021). The specimen was first identified as a *Mylodon armatus* Lydekker, 1887 by Schulthess (1920), and, following literature, this species was synonymized with *L. armatus* (Czerwonogora & Fariña, 2013). As with Neosclerocalyptinae, I propose to follow the synonymization at the genus level and reassign PIMUZ A/V 503 to *Lestodon* sp.

*Glossotherium* Owen, 1839a

*Glossotherium robustum* (Owen, 1842)

**Referred material:** Cranium, dentary, 16 vertebrae (cervical, thoracic, and caudal), 25 postcranial elements (including ribs and phalanges), and four plates with osteoderms: PIMUZ A/V 484 (Figs. 3, 10, 11); cranium: PIMUZ A/V 485; right upper fourth molariform: PIMUZ A/V 486; right upper molariform: PIMUZ A/V 487; lower third molariform: PIMUZ A/V 488; left third lower molariform: PIMUZ A/V 497; right first lower molariform: PIMUZ A/V 498; right third lower molariform: PIMUZ A/V 499; left fragmented tibia: PIMUZ A/V 501; eight bones of the foot: PIMUZ A/V 502 (Fig. 7); incomplete right mandible: PIMUZ A/V 4143; fragmented cranium in about 50 pieces: PIMUZ A/V 4144.

**Comment**: The Mylodontinae have been the subject of nomenclatural confusion for a long time, particularly with respect to *Glossotherium* and *Mylodon* (see De Iuliis et al., 2017; McAfee, 2009). Many recent studies have attempted to improve the diagnoses of multiple mylodont species (e.g., Boscaini et al., 2020b; Brambilla & Ibarra, 2018a; McAfee, 2009, 2016; Pitana et al., 2013). Initial diagnoses to distinguish mylodont species were based primarily on cranial characters including teeth (Brambilla & Ibarra, 2018a; McAfee, 2009). For *Glossotherium*, at least four species are recognized in South America, but only *Gl. robustum* is known from the Pleistocene of the Pampean formation associated with the presence of *Glossotherium* sp. (Pitana et al., 2013). In the Roth collection at PIMUZ, four specimens have cranial remains. PIMUZ A/V 484 and PIMUZ A/V 485 are by far the most complete specimens. The former shows a dental formula of 5/5 allowing to distinguish it from this specimen from *M. darwinii* (McAfee, 2009). In cross section, the upper caniniform is triangular, the Mf1 and Mf2 have a similar mesiodistal length, and the Mf4 is bilobate with a posterior lobe mediolaterally narrower than the anterior lobe. Unfortunately, the upper dentition of PIMUZ A/V 485 is not preserved. However, both specimens possess diagnostic cranial characters allowing to reject an assignment to *M. darwinii* and support an attribution to *Gl. robustum* (McAfee, 2009), including a short palatal length posterior to Mf4, a dome-shaped cranium with its maximum high at the level of the postorbital processes, narrow snout in front of the anterior margin of the orbit, and width of the nasal cavity greater than its height. PIMUZ A/V 484 and PIMUZ A/V 485 differ from *Archaeomylodon sampedrinensis* Brambilla & Ibarra, 2018a, in having a less subcircular occiput and the absence of a prominent diastema between the Cf1 and Mf1 (Brambilla & Ibarra, 2018a). Specifically, the snouts of these specimens show the absence of a nasal elevation and well-marked fossa for the muscle buccinator, both features supporting their assignment to *Gl. robustum* rather than to *A. sampedrinensis* (Brambilla & Ibarra, 2018a). I therefore propose to reassign these specimens to *Gl. robustum*. PIMUZ A/V 4144 is far too fragmentary to ensure a clear identification. The little identifiable material is not different from the most complete specimens and suggests a reassignment to *Gl. robustum* rather than *M. darwinii*. The mandible of *A. sampedrinensis* is still unknown, and that of *M. darwinii* has the particularity of having a predental spout longer than the length of the tooth row. PIMUZ A/V 4143 shows a relative length of the tooth row longer than the predental spout, as in the mandible of PIMUZ A/V 484, which leads me to favor a reassignment to *Gl. robustum*. On the basis of the skull of PIMUZ A/V 484, I have evaluated the identification of the specimens represented only by isolated teeth. PIMUZ A/V 486 has a general shape closer to Mf1 than to Mf4, despite its initial determination (see Additional file 1: Table S1), but sulci are visible on either side of the most distal part of the tooth, suggesting that the tooth is an Mf4. PIMUZ A/V 486 was probably a young specimen, implying that tooth lobation occurred progressively during tooth eruption and the early stages of tooth wear. This is a hypothesis to be explored that I suggest for both sloths and glyptodonts, mammalian groups with hypso/hypselodonty and lobation (Bargo et al., 2006b; Vizcaíno et al., 2011). Because of its relatively young ontogenetic stage, a clear determination for PIMUZ A/V 486 is difficult to establish. However, the specimen perfectly matches the morphology of the Mf4 of a young *Gl. robustum* (Pitana et al., 2013). Therefore, I propose to assign PIMUZ A/V 486 to *Gl. robustum* while noting that this determination remains fragile. In contrast, PIMUZ A/V 487 exhibits an Mf4 bilobate with a posterior lobe narrower than the anterior lobe, suggesting an assignment to a small *Gl. robustum*. For the lower teeth, PIMUZ A/V 488 was identified as an mf3 but it does not show bilobation. It is therefore more likely that this tooth corresponds to an mf2. The relatively subtriangular shape of the tooth suggests an attribution to *Gl. robustum,* as for PIMUZ A/V 497 and PIMUZ A/V 499, but these reassignments need to be considered with caution. PIMUZ A/V 498 corresponds to a well-developed caniniform compatible with *Gl. robustum* (see Bargo & Vizcaíno, 2008). Regarding the postcranial material, the recent work of McAfee (2016) allowed further characterizations of *M. darwinii* and *Gl. robustum*, but no postcranial material was described for *A. sampedrinensis*. Unfortunately, the description of the foot and pelvic girdle is not included in the diagnoses of *M. darwinii* and *Gl. robustum* (Cartelle et al., 2019; McAfee, 2009, 2016). PIMUZ A/V 502 corresponds to eight bones of the foot including the calcaneum, cuboid, metatarsus IV, and metatarsus V. The portion of the foot preserved for this specimen matches with the full foot drawing proposed by Schulthess (1920) for *Mylodon robustus* Owen, 1842, a synonym of *Gl. robustum* (see McAfee, 2009), and the shape of the calcaneum is relatively close to that of other *Glossotherium* species (e.g., Cartelle et al., 2019). Finally, PIMUZ A/V 501 shows a distal articular portion closer to *Gl. robustum* (see Cartelle et al., 2019) than to *M. darwinii*, as the medial portion of the astragalar articulation (= odontoid process facet in Boscaini et al., 2021) is relatively deeper, but mainly because this portion is well separated from the lateral portion for the articulation with the distal fibula (= distal fibular facet in Boscaini et al., 2021) according to the diagnosis of *M. darwinii* provided by McAfee (2016) (see also Roth, 1899). In the Roth collection at PIMUZ, the skeleton of *Gl. robustum* is relatively well represented. A particular feature of the collection is the presence of four association of osteoderms belonging to PIMUZ A/V 484, providing an opportunity to explore osteoderm development in xenarthrans (e.g., McDonald, 2018; Toledo et al., 2021).

*Archaeomylodon* Brambilla & Ibarra, 2018a

*Archaeomylodon sampedrinensis* Brambilla & Ibarra, 2018a

**Referred material:** Three cranial fragments, one corresponding to the left posterior part of the basicranium and undetermined: PIMUZ A/V 4140 (Fig. 10).

**Comment**: of the three fragments available for PIMUZ A/V 4140 do not bear diagnostic elements, while the third fragment corresponds to a part of the occiput. The latter exhibits a well-marked occipital condyle of similar size to that of *Lestodon* (see above). The preserved part of the occiput seems to induce a complete subcircular shape of the occipital region, which is more consistent with *A. sampedrinensis* than with other Mylodontinae (Brambilla & Ibarra, 2018a). The most diagnostic feature is indicated by the distance between the occipital condyle and the hypoglossal foramen. Similar to *A. sampedrinensis*, this distance is almost twice the distance found in other Mylodontinae (Brambilla & Ibarra, 2018a). I therefore propose the reassignment of PIMUZ A/V 4140 to *A. sampedrinensis*. While the specimen is particularly incomplete, PIMUZ A/V 4140 has the major interest of being one of the few known occurrences of this mylodont in the Pampean Region.

*Mylodon* Owen, 1839a

*Mylodon darwinii* Owen, 1839a

**Referred material:** Left fourth lower molariform: PIMUZ A/V 495; cranial vault and fragments: PIMUZ A/V 496 (Fig. 10); incomplete left mandible: PIMUZ A/V 504 (Fig. 11); incomplete left mandible: PIMUZ A/V 505; right tibia: PIMUZ A/V 4147 (Fig. 6).

**Comment**: The genus *Mylodon* is considered as monospecific since the work of Esteban (1996) although a recent study suggests that a Patagonian species attributed to this genus probably existed (Brambilla & Haro, 2022). The only cranial remains belonging to *M. darwinii* in the PIMUZ Santiago Roth Collection correspond to the cranial vault and the dorsal part of the occiput. These cranial regions are frequently found in ground sloths, which led Brambilla and Ibarra (2018b) to produce a study focused on the occiput. While occipital shape has been shown to be a complex character within mylodonts (see Boscaini et al., 2022; De Iuliis et al., 2020;), PIMUZ A/V 496 does not exhibit the occiput enlargement known in *Gl. robustum* compared to other sloths (Brambilla & Ibarra, 2018b). Although the occiput is not complete, a lateromedially subelliptical rather than subcircular shape is observed in the specimen, which would favor an attribution to *Gl. robustum* (Brambilla & Ibarra, 2018b). The specimen is relatively small in size for each of the potential species and the nuchal crests are only weakly developed. In particular, Brambilla and Ibarra (2018b) proposed that the weak development of the nuchal crests corresponds to an ontogenetic character suggesting that their protrusion occurred towards the end of ontogeny. These same authors explain that the occiput of *M. darwinii* also tends to become more subcircular towards the end of growth and, therefore, that subadult specimens can be confused with *Gl. robustum* if one does not consider variation associated with ontogenetic change. Therefore, I suggest that PIMUZ A/V 496 could correspond to a subadult representative of *M. darwinii*. PIMUZ A/V 495 corresponds to the anterior lobe of an mf3 with a particularly narrow connection to the posterior lobe, in contrast to *Gl. robustum* (Bargo & Vizcaíno, 2008). Although the connection between the lobes of the mf3 is not a sufficiently diagnostic element to discern between the species (McAfee, 2009), I suggest to keep the attribution to the genus *Mylodon* and thus reassign PIMUZ A/V 495 to *M. darwinii*. The same shape for mf3 is observed in PIMUZ A/V 504, but identification of the specimen is limited by the quality of preservation of the mandible and teeth. The shape of the predental spout differs between *Gl. robustum* and *M. darwinii* (see above) but this anterior region is not preserved in PIMUZ A/V 504 and PIMUZ A/V 505. For these specimens, the general shape of the dental alveoli does not allow to reject an attribution to the genus *Mylodon*, suggesting an assignment to *M. darwinii*. I note, however, that PIMUZ A/V 505 is much smaller than PIMUZ A/V 504 and that the lobation of the teeth is only slightly advanced, suggesting that PIMUZ A/V 505 is a juvenile or subadult specimen. Finally, PIMUZ A/V 4147 is a subcomplete tibia in which the lateral portion for articulation with the distal fibula is almost not preserved. The contact of this portion with the medial portion of the astragalar articulation is nevertheless visible and forms an obtuse edge as in *M. darwinii* (McAfee, 2016), although the medial portion of the astragalar articulation is not particularly shallower than the specimen previously identified as *Gl. robustum*, *i.e*., PIMUZ A/V 501. I therefore propose a reassignment of PIMUZ A/V 4147 to *M. darwinii*. Darwin's *Mylodon* is poorly represented in Roth collection at PIMUZ, but the species has the particularity of including juvenile representatives, allowing to measure the ontogenetic variation on the mandible. The presence of a well-preserved tibia of good preservation quality is also noteworthy, and is potentially useful for functional studies (e.g., Toledo et al., 2012).

*Scelidotheriidae* Ameghino, 1889

*Scelidotheriinae* Ameghino, 1904

Indet.

**Referred material:** Right jugular and dorsal vertebra: PIMUZ A/V 514; mandible fragment bearing four broken teeth: PIMUZ A/V 518; incomplete cranium and one astragalus: PIMUZ A/V 526; altered anterior part of the cranium: PIMUZ A/V 527; six caudal vertebrae: PIMUZ A/V 529; one radius: PIMUZ A/V 530; one foot bone: PIMUZ A/V 4104; one ulna: PIMUZ A/V 4134; rostrum bearing teeth and a part of the mandible: PIMUZ A/V 5699.

**Comment**: The Scelidotheriinae are by far the most abundant group in the Roth collection at PIMUZ. Following multiple revisions on the diversity of the ‘subfamily’, only three genera are recognized in the Pleistocene: *Scelidotherium* Owen, 1839a, *Catonyx* Ameghino, 1891, and *Valgipes* Gervais, 1873 (e.g., Miño-Boilini & Carlini, 2009; Miño-Boilini & Quiñones, 2020). Among them, four species are known to occur in the Pampean Region: *Scelidotherium leptocephalum* Owen, 1839a, *Scelidotherium bravardi* Lydekker, 1886, *Catonyx cuvieri* Lund, 1839, and *Catonyx tarijensis* Gervais & Ameghino, 1880 (Miño-Boilini & Quiñones, 2020). The Scelidotheriinae group now has extensive diagnoses including cranial, dental, and postcranial diagnostic features (e.g., Miño-Boilini et al., 2014), which helps to avoid confusion with other mylodontines. Specific identification, however, focuses primarily on variations in dentition and autopod traits (e.g., Corona et al., 2013; Nieto et al., 2021). Identification of PIMUZ A/V 5699 is only possible at ‘subfamilial’ rank due to the early ontogenetic stage of the specimen. Because of the presence of laterally compressed molariforms and a relatively elongated cranium, an attribution to the Scelidotheriinae is the most likely (Miño-Boilini et al., 2019). PIMUZ A/V 514, a young specimen, and PIMUZ A/V 529 are present only through the preservation of caudal vertebrae and a jugal (PIMUZ A/V 514), parts of the skeleton that do not provide particularly diagnostic features. PIMUZ A/V 526, PIMUZ A/V 530, PIMUZ A/V 4104, and PIMUZ A/V 4134 correspond to an altered cranium, long bones, and foot bones for which the only potential diagnostic information corresponds to the relatively large size of these specimens, a fragile argument for this clade. Finally, PIMUZ A/V 518 and PIMUZ 527 are far too altered to distinguish diagnostic traits. Because of the alteration of these specimens, their relatively young age or the absence of diagnostic elements, I favor an attribution to the ‘subfamily’ level.

*Scelidotherium* Owen, 1839a

*Scelidotherium leptocephalum* Owen, 1839a

**Referred material:** Skull, atlas, five vertebrae, scapula, radius, ulna, femur, tibias, fibulae, foot bones, humerus, one complete hand, two isolated hand bones, patella, and ribs: PIMUZ A/V 508 (Figs. 5, 6, 7); skull, right humerus, one radius, two femurs, two fibulae, one rib, and six caudal vertebrae: PIMUZ A/V 509 (Fig. 11); cranium and one phalanx bearing claw: PIMUZ A/V 510; skull (including ear ossicles), isolated teeth, vertebrae, ribs, two clavicles, left scapula, and right humerus: PIMUZ A/V 513 (Fig. 10); upper jaw fragment bearing four broken teeth: PIMUZ A/V 515; anterior part of the mandible: PIMUZ A/V 517; jaw fragment: PIMUZ A/V 521; two caniniforms and one mf1 or mf2: PIMUZ A/V 522; one tibia and one finger: PIMUZ A/V 531; seven postcranial bones: PIMUZ A/V 532; mandible and many postcranial bones: PIMUZ A/V 4128; maxillary bearing left Mf1 and Mf2: PIMUZ A/V 4130; right posterior half of the cranium and right mandible: PIMUZ A/V 4149.

**Comment**: The species *S. leptocephalum* was originally the assignment proposed by Roth (1889) and Schulthess (1920) for most of the scelidothere specimens at PIMUZ collection. The present study confirms the greater abundance of this species relative to other scelidotheres, although several specimens were reassigned to other species (see below). The completeness of the specimens ranges from almost complete skeletons to isolated tooth fragments, but several specimens are particularly well-preserved. PIMUZ A/V 508, PIMUZ A/V 509, PIMUZ A/V 510, and PIMUZ A/V 513 include well-preserved craniodental remains and all the specimens preserve the dentition. The attribution to the genus *Scelidotherium* is confirmed by the presence of a relatively narrow nasal cavity, an almost straight cranial roof in lateral view, an elongated premaxilla, and teeth of relatively small size compared to the size of the cranium (Miño-Boilini et al., 2014). For the two specimens with at least one femur preserved, PIMUZ A/V 508 and PIMUZ A/V 509, assignment to the genus *Scelidotherium* is also supported by short femora possessing a medial inclination of the proximal end relative to the distal end and a concave medial surface of the femoral stem (Miño-Boilini et al., 2014). The distinction between the two species of *Scelidotherium* found in the Pampean Region, i.e., *S. leptocephalum* and *S. bravardi*, is more complex. *S. leptocephalum* is recognized as a large species present mainly in the late Ensenadan, Bonaerian and Lujanian while *S. bravardi* is significantly smaller in size and is mainly recovered in the Ensanadan (Miño-Boilini & Quiñones, 2020; Miño-Boilini et al., 2014). For PIMUZ A/V 508, PIMUZ A/V 509, and PIMUZ A/V 513, assignment to *S. leptocephalum* is supported by their large size, narrow anterior predental region of the mandibular ramus more ventral than in *S. bravardi*, elliptical Cf1 with a slight lingual lobe, and upper teeth without apicobasal sulci (Miño-Boilini et al., 2014). Also, the humeri show a double entepicondylar foramen, a feature that was included in the diagnosis of *S. bravardi* (Miño-Boilini et al., 2014), and also of *Ca. tarijensis*, but not in *Ca. cuvieri* (Miño-Boilini, 2016). The presence of the double foramen on the humerus seems to represent a variable trait, but not a diagnostic character. I therefore prefer to retain the original attribution for these three specimens to *S. leptocephalum*. For PIMUZ A/V 510, the teeth are heavily altered but the cranium of the specimen is large and, in all respects, similar to the *S. leptocephalum* illustrated in the study by Miño-Boilini and Quiñones (2020; Fig. 3B—FMNH P 14,294) and consequently referred to this species. For six specimens, only the dentition features provide specific information. PIMUZ A/V 515 exhibits the upper dentition from Mf1 to Mf4, in particular the shape of Mf2 and Mf4 correspond to elongated triangles without grooves and without distinctive lobes in occlusal view. This tooth morphology together with the relatively large size supports the assignment to *Scelidotherium* (Corona et al., 2013; Miño-Boilini et al., 2014). Comparison with the tooth pattern presented by Corona et al. (2013) supports the same conclusion for the upper and lower dentition of PIMUZ A/V 517, PIMUZ 521, PIMUZ A/V 522, PIMUZ A/V 4149, and PIMUZ A/V 4130. For specimens with mf3, such as PIMUZ A/V 521, I note that the tooth does not show a straight posterior lobe but a C-shaped curve in occlusal view, as recognized in *S. leptocephalum* and unlike *S. bravardi* (Miño-Boilini et al., 2014). Three specimens are distinguishable only on the basis of postcranial elements. PIMUZ A/V 531 show a metacarpus, probably the II, and a tibia very similar to those of PIMUZ A/V 508. The same conclusion emerges from the comparison of the autopodial bones and the tibia of PIMUZ A/V 532 with PIMUZ A/V 508. On the other hand, PIMUZ A/V 4128 exhibits several isolated elements of the autopodium, including bones of the hand, but considering its small size and the stage of the tooth eruption from the mandible, the specimen is young or at least subadult, limiting comparisons with the broad description of adult specimens in the study of Nieto et al. (2021). As there are no different features compared to the previously mentioned specimens except for a smaller size, I prefer to retain the original attribution to *S. leptocephalum*. The presence of several ontogenetic stages, with PIMUZ A/V 4128 and PIMUZ A/V 4130 being particularly young specimens, and the abundance of *S. leptocephalum* highlights the significance of the Roth collection at PIMUZ.

*Scelidotherium bravardi* (Lydekker, 1886)

**Referred material:** Cranial fragment, hemimandible, five ribs, one cervical vertebra, fragment of vertebral apophysis, right femur and foot, left scapula, femoral head, tibia, and pelvic fragment: PIMUZ A/V 506 (Figs. 6, 11); incomplete maxilla bearing the right Cf1 and almost complete right mandible with complete dentition: PIMUZ A/V 507; caniniform, mf1, and mf2: PIMUZ A/V 519; left mf2: PIMUZ A/V 520; five caudal vertebrae and several bones of the foot: PIMUZ A/V 528 (Fig. 7); incomplete right mandible: PIMUZ A/V 4093.

**Comment**: The specimens reassigned here to *S. bravardi* all appear to be adult and relatively smaller in size than those previously treated for *S. leptocephalum.* The only exception is represented by PIMUZ A/V 507, a young specimen in which the predental spout is oriented more dorsally than in *S. leptocephalum*, a diagnostic feature of *S. bravardi* (Miño-Boilini et al., 2014). In addition, the posterior lobe of the mf3 is straight, without the characteristic C-shaped cross-section of *S. leptocephalum* (Miño-Boilini et al., 2014). PIMUZ A/V 506 shows the same characteristics in the mandible. The mf3 of PIMUZ A/V 506 and PIMUZ A/V 4093 also exhibits the same shape of PIMUZ A/V 507 in occlusal view. I therefore propose a reassignment to *S. bravardi* for these three specimens. PIMUZ A/V 519 and PIMUZ A/V 520 have the tooth pattern proposed by Corona et al. (2013) for *Scelidotherium* but with a strong trilobulation and an elliptical caniniform in cross-section (PIMUZ A/V 519) which suggests a reassignment to *S. bravardi* (Miño-Boilini et al., 2014). Only one specimen can be identified based on postcranial material: PIMUZ A/V 528. Among multiple foot bones, PIMUZ A/V 528 has a well-preserved calcaneum for which the sustentacular facet is slightly continuous with the cuboid facet in proximal view, a feature favoring a reattribution to *S. bravardi* (Miño-Boilini et al., 2014). The presence of *S. leptocephalum* and *S. bravardi* in the Pampean Region offers the opportunity to relate the evolution of the clade with the drastic paleoenvironmental variations of the region during the Pleistocene, especially since *S. leptocephalum* is considered a species particularly well adapted to arid environments (see below). The high abundance of both species in the Roth collection at PIMUZ will allow to generate comparisons on intraspecific variation between the two species. These occurrences reveal the evolutionary success of the clade in the Pleistocene Pampean Region with a much higher abundance of *S. leptocephalum* than *S. bravardi*.

*Catonyx* Ameghino, 1891

*Catonyx tarijensis* (Gervais & Ameghino, 1880).

**Referred material:** Cranium: PIMUZ A/V 511; cranium: PIMUZ A/V 512 (Fig. 10); mandible fragment with three teeth: PIMUZ A/V 516; Mf1: PIMUZ A/V 524; Mf1: PIMUZ A/V 525; mandible fragment: PIMUZ A/V 4129.

**Comment**: This generic reassignation is largely based on the study of PIMUZ A/V 512, an exceptionally well-preserved cranium. PIMUZ A/V 512 exhibits a mediolaterally wider nasal cavity than in *Scelidotherium*, a convex cranial roof in lateral view, a relatively more robust dentition than *Scelidotherium*, a mediolaterally broad snout, and a mesiodistally elongated caniniform. These morphological traits suggest reassignment to the genus *Catonyx* (following Miño-Boilini et al., 2014) rather than confirming the initial assignment to *Scelidotherium* (see Additional file 1: Table S1). For a more specific determination, the dentition is the main diagnostic element (Corona et al., 2013; Miño-Boilini, 2016). PIMUZ A/V 512 shows a weak trilobulation of Mf1 and Mf2 without a prominent groove, supporting an assignment to *Ca. tarijensis* rather than *Ca. cuvieri* (Corona et al., 2013; Miño-Boilini, 2016). The occlusal shape pattern of PIMUZ A/V 512 matches that proposed by Corona et al. (2013) for *Ca. tarijensis* (see Fig. 5C—MMP n/n) and it is here assigned to this species*.* PIMUZ A/V 511 has a cranium similar to PIMUZ A/V 512 and this is the basis for the taxonomic interpretation; although teeth are not preserved. PIMUZ A/V 516 shows a less trilobate shape in occlusal view from mf2 to mf4, while mf4 has a triangular shape as reported for Ca. *tarijensis* species by Corona et al. (2013). PIMUZ A/V 524, PIMUZ A/V 525, and PIMUZ A/V 4129 were all originally assigned to *Scelidotherium patrium* Ameghino, 1888, a species that is now invalid and considered juveniles of *Ca. tarijensis* (Miño-Boilini et al., 2019). The presence of *Catonyx* in the Roth collection at PIMUZ brings a new complexity regarding the diversity of scelidotheres, as *Ca. tarijensis* is considered a species with a high ecological tolerance to different environments and altitudes (Miño-Boilini & Quiñones, 2020). A more important fact is the high preservation quality of two subcomplete crania in the collection although the cranial and endocranial anatomy of this species was already well described (Boscaini et al., 2020a; Miño-Boilini, 2016).

*Catonyx cuvieri* (Lund, 1839)

**Referred material:** Right mf1, mf2, mf4, Mf1, Mf3, and left Mf1: PIMUZ A/V 523.

**Comment**: PIMUZ A/V 523 corresponds to a specimen for which six isolated teeth were preserved. There is a weak trilobulation of Mf1 and a strong trilobulation of mf1 that suggest the reassignment of this specimen to *Catonyx* (see Corona et al., 2013). Mf3 from PIMUZ A/V 523 shows two strongly marked lobes with a thin connection between them, with the posterior having a straight distal elongation. The mf4 of PIMUZ A/V 523, on the other hand, exhibits a less trilobated shape, and is more rounded than triangular. In agreement with the dental patterns depicted by Corona et al. (2013), all of these traits of the Mf3 and mf4 suggest a reassignment of PIMUZ A/V 523 to *Ca. cuvieri*. This species is considered as a ground sloth species living in humid habitats such as tropical and subtropical forests (Miño-Boilini & Quiñones, 2020). The presence of this species contrasts with the abundance of *S. leptocephalum* and indicates a likely complex environmental scenario of the Pampean Region.

*Megatheriidae* Gray, 1821

*Megatheriinae* Gray, 1821

*Megatherium* Cuvier, 1796

*Megatherium americanum* Cuvier, 1796

**Referred material:** Fragmented mandible: PIMUZ A/V 478; almost complete skeleton: PIMUZ A/V 479 (Fig. 1); one tooth, one right phalanx, and two caudal vertebrae: PIMUZ A/V 481; radius, ulna, two complete hands: PIMUZ A/V 482 (Fig. 7); one radius: PIMUZ A/V 483 (Fig. 5).

**Comment**: *Megatherium* is probably the most famous giant sloth, perhaps both because of its gigantic size and for the historical context of its first descriptions by George Cuvier (1796) (Argot, 2008). According to De Iuliis (1996), only the species *M. americanum* lived in the Pampean Region, contrary to the supposed diversity of this genus (see Agnolin et al., 2018; and citation therein). Brandoni et al. (2008) restored the validity of the species *M. gallardoi* Ameghino & Kraglievich, 1921, stating that this species is commonly recovered in the Ensenadan while *M. americanum* occurs primarily in the Lujanian. The most recent work on *Megatherium* from the Pampean Region supports the validity of at least two species, *M. americanum* and *Megatherium filholi* Moreno, 1888 (Agnolin et al., 2018). The distinction between the two previously cited species is primarily a difference in size, with *M. filholi* being much smaller (femoral length smaller than 60 cm) and more gracile than *M. americanum* (Agnolin et al., 2018). Considering that the diagnoses of *M. filholi* is based mainly for femur characters, PIMUZ A/V 479, has a femur greater than 60 cm in height, greater trochanter higher than the femoral head, and patellar trochlea not medially extended, that suggest an attribution to *M. americanum* over *M. filholi* (Agnolin et al., 2018). The criterium of using size as a diagnostic feature by Agnolin et al. (2018) is problematic in the case of a small specimen with respect to ontogenetic and individual variation, or potential dimorphism as it has been reported in other sloths (e.g., Boscaini et al., 2019; Cartelle & De Iuliis, 1995). PIMUZ A/V 478, PIMUZ A/V 481, PIMUZ A/V 482, and PIMUZ A/V 483 are all large specimens similar to the subcomplete skeleton of PIMUZ A/V 479, suggesting an attribution to *M. americanum*.

*Megatherium* sp.

**Referred material:** One metacarpal bone, and three cranial fragments including the premaxilla, a descending process of the jugal, and an incomplete right neurocranium including the middle ear ossicles: PIMUZ A/V 480 (Fig. 10).

**Comment**: PIMUZ A/V 480 appears to be slightly smaller in size than the cranium of PIMUZ A/V 479 but its fragmentary condition prevents from reaching solid conclusions. The descending process of the jugal of this specimen presents a less mediolaterally flattened shape at the tip of the process, in contrast to *M. americanum*. Despite this minor difference, potentially impacted by taphonomy, I prefer to assign PIMUZ A/V 480 to *Megatherium* sp. pending a complete revision of the genus.

*Nothrotheriidae* Gaudin, 1994

*Nothrotheriinae* Ameghino, 1920

*Nothrotherium* Lydekker, 1889

*Nothrotherium escrivanense* (Reinhardt, 1878)

**Referred material:** Cranium, seven thoracic vertebrae, caudal vertebrae, ribs, sternum, humerus, radius, ulna, three hand bones: PIMUZ A/V 477 (Figs. 5, 6, 10).

**Comment**: Nothrotheres are particularly well-known in the northern part of the Pampean Region, especially in the southern part of Santa Fe Province (Vezzosi et al., 2019). There are at least four species from the late Pleistocene of the Pampean Region: *Nothropus carcaranensis* Bordas, 1942; *Nothropus priscus* Burmeister, 1882*; Nothrotherium torresi* Kraglievich, 1926, and *Nothrotherium escrivanense*. Only one specimen in the Roth collection at PIMUZ corresponds to a nothrothere according to the work of Schulthess (1920). She attributed the almost complete specimen PIMUZ A/V 477 to the species *No. escrivanense*. Subsequently, several authors (e.g. Cartelle & Fonseca, 1983; Paula Couto, 1971; Perea, 2007) have proposed that *No. escrivanense* corresponds to a juvenile of *No. maquinense* (Lund, 1839) although Pujos (2001) considered the species to be valid. A revision of the taxonomic diversity of nothrotheres in the Pampean Region should be carried out (Brandoni & Vezzosi, 2019). PIMUZ A/V 477 follows perfectly the diagnosis of the genus proposed by Pujos (2001), with a small size, cylindrical and elongated cranium, distinct area for prominent pterygoid sinuses, globular parietal region higher than the frontal and exhibiting strong posterior inclination, absence of caniniform, anteroposteriorly narrow Mf4, that appear less quadrangular compared to the other teeth based on the tooth alveoli. The femur also has diagnostic elements, but this bone was not found for PIMUZ A/V 477. Specifically, the diagnosis of *No. escrivanense* is mainly focused on the upper teeth and several features on the hand and foot (Pujos, 2001) that are absent in PIMUZ A/V 477 except for the right Mf1. Based on the dental alveoli, the molariforms appear to be weakly mesiodistally compressed and the mesiodistal diameter between the labial and lingual faces of Mf1 to Mf3 shows little difference which supports the validity of the attribution to *No. escrivanense* (Pujos, 2001). Pending a review of the diversity of nothrotheres from the Pampean Region, I propose to retain the attribution proposed by Schulthess (1920). In addition to the completeness of the specimen, PIMUZ A/V 477 is of major interest for the reassessment of the diversity of Pleistocene nothrotheres from Argentina. The quality of preservation would also allow a future examination of its endocranial structures. The specimen includes several well-preserved long bones (see above) as partially documented in histological analyses (Houssaye et al., 2016a, b; Straehl et al., 2013).

### Diversity and Abundance of Santiago Roth's Xenarthrans stored in the PIMUZ

**Fig. 12:**
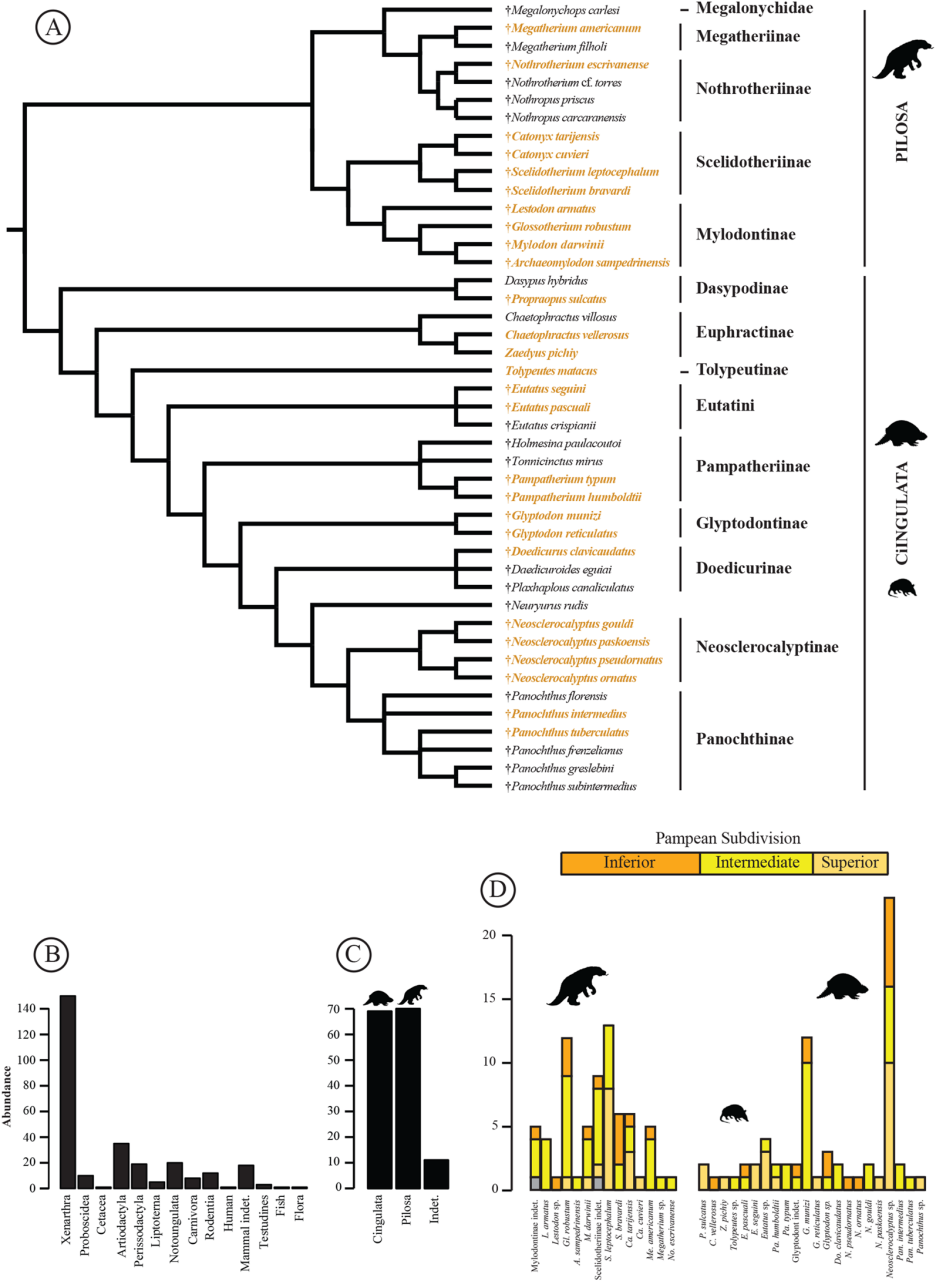
Diversity and abundance of the xenarthrans from the Roth collection at PIMUZ. **A** Reconstructed phylogeny of xenarthrans found in the Pampean Region during the Pleistocene (see text for references). The species referenced in the Roth collection at PIMUZ are colored. **B** Abundance at the scale of vertebrates specified as the number of specimens per taxon in the collection. **C** Abundance of xenarthrans specified as the number of specimens per order in the collection. **D** Abundance of each xenarthran species specified as the number of specimens per species. The colors follow those of the Pampean subdivisions (see Fig. 2)

Among the 284 specimens in the Roth collection at PIMUZ, 150 belong to Xenarthra (~ 53%) mainly collected in the northern part of the Pampean Region (Fig. 2). This high abundance of xenarthrans replicates the pattern already highlighted by many researchers in the Pleistocene Pampean faunas compared to other mammals (e.g., Carlini & Scillato-Yané, 1999; Cione et al., 1999; Soibelzon & Tonni, 2009; Soibelzon et al., 2010; Tonni et al., 1999a, b). The present taxonomic reassessment also revealed a high taxonomic diversity in the Roth collection (Figs. 2, 12). Among all the xenarthrans studied in the present investigation, 69 specimens belong to the Cingulata (46%), 70 specimens belong to the Folivora (~ 47%), and 11 (7%) correspond to undetermined or lost material. As such, Cingulata and Folivora are both equally abundant in the Roth collection at PIMUZ, with a higher diversity for Cingulata (between 17 and 21 sp.) than for Pilosa (between 10 and 12 sp.) (Fig. 12). Among the Cingulata, three specimens belong to three extant species of armadillos (~ 4%) (Gibb et al., 2016). With the inclusion of *P. sulcatus*, the Roth collection at PIMUZ contains at least three of the four extant armadillos ‘subfamilies’ (Fig. 12) (Gibb et al., 2016). The diversity of small-sized armadillos is low in the Roth collection at PIMUZ compared to that of the extinct large-sized armadillos. Among them, the Eutatini are represented by eight of the smallest specimens (~ 12%) distributed in two species. However, large-sized pampatheres that can reach ~ 200 kg (McDonald, 2005) are also represented by few (i.e., four) specimens, listed in two species (~ 6%). This under-representation of pampatheres in the Pampean Region was already mentioned by previous works (Carlini & Scillato-Yané, 1999; Cione et al., 1999; Soibelzon et al., 2010; Tonni et al., 1999a, b). It is possible that because of their large size and herbivorous diet, the distribution of pampatheres was affected by the exceptional proliferation of giant extinct armadillos, the glyptodonts, the most abundant clades of cingulates in the Pampean Region (e.g., Carlini & Scillato-Yané, 1999; Cione et al., 1999; Tonni et al., 1999a, b; Soibelzon & Tonni, 2009; Soibelzon et al., 2010), with 52 specimens present in the Roth collection at PIMUZ (~ 75%—proportion of glyptodonts among cingulates in the collection). Glyptodonts are represented by at least nine species, with one representative of each of the four ‘subfamilies’ of Pleistocene glyptodonts. The abundance of each glyptodont ‘subfamilies’ in the collection follows a size gradient. Neosclerocalyptinae, small glyptodonts of about ~ 450 kg (Vizcaíno et al., 2011), are represented by 28 specimens (~ 41%), with at least one representative of each known Pleistocene Neosclerocalyptinae species (Zurita et al., 2011). In the intermediate gigantic sizes, about one to two tons (Fariña et al., 1998), the second most abundant ‘subfamily’ corresponds to the Glyptodontinae, with 16 specimens (~ 23%) indicating the presence of two of the three species of the genus *Glyptodon*. Except for the species *G. jatunkhirkhi*, a large part of the diversity of Glyptodontinae from the southern part of South America are represented in the collection (Zurita et al., 2018). Also gigantic in size (Fariña et al., 1998), the third ‘subfamily’, Panochthinae, is particularly scarce in the collection with four specimens (~ 6%), represented by two species of the genus *Panochthus*. Recently, Brambilla et al. (2020) highlighted a high diversity for this genus in the Pampean Region with six species. With respect to the Panochthinae, the Roth collection at PIMUZ has a relatively low diversity and abundance for this clade, with an over-representation of the species from the Ensenadan. Finally, with the large *Doedicurus*, up to three tons (Fariña et al., 1998), the Doedicurinae is represented by only two specimens in the collection (~ 3%), including the only doedicurine species known in the Pleistocene (Soibelzon et al., 2010). The Folivora is less diversified and is represented only by ground sloth specimens through four ‘subfamilies’: Mylodontinae, Scelidotheriinae, Megatheriinae, and Nothrotheriinae. The Roth collection at PIMUZ is mainly composed of mylodontines and scelidotheriines. The former is represented by 28 specimens (40%) while the latter is represented by 35 specimens (50%). These two ‘subfamilies’ represent giant sloths of gigantic size (Fariña et al., 1998). In contrast, the two ‘subfamilies’ with the most extreme sizes, for the smallest, the nothrotheres with a body mass of more than 171 kg (Dantas, 2022), and for the largest, the megatheres with an extreme bodymass estimated between three and six tons (Fariña et al, 1998), are only marginally present in the Roth collection at PIMUZ, with only one specimen for nothrotheres (~ 1%) and six specimens for megatheres (~ 9%). There were likely 44 valid species of xenarthrans in the Pleistocene of the Pampean Region (see Brambilla & Ibarra, 2018a; Carlini & Scillato-Yané, 1999; Cione et al., 1999; Krmpotic et al., 2009; Tonni et al., 1999a, b) (Fig. 12). With its at least 27 species of xenarthrans, the Roth collection at PIMUZ includes more than half of the known species of xenarthrans from the Pleistocene of the Pampean Region.

### Contribution to the reconstruction of the Pampean Region paleoenvironment

The Pleistocene is a period marked by a large temperature amplitude associated with a greater frequency of glacial and interglacial periods (e.g., Prado et al., 2021; Sanz-Pérez et al., 2022; Soibelzon, 2019). This epoch was therefore subject to drastic changes oscillating rapidly between cold and warm periods. Climatic variations affected the environments, triggering abiotic and biotic events of ample magnitude while causing a multitude of retroactive phenomena (see Prado et al., 2021). Some regions were more prone to suffer these drastic climatic variations. Bordered by the subtropical forest at the north and the Patagonian arid environment at the south, the Pampean Region was one of these regions. Therefore, the Pampean Region is cyclically transformed by the southward extent of wet environments in interglacial periods and the northward extent of arid environments in glacial periods (Quattrocchio et al., 2008; Tonni, 2017). The environment of the Pampean Region during the Pleistocene was therefore continuously transformed and impacted by changes in the flora sometimes towards grassy steppes in cold periods, and tree savannas or forests in warm periods (Quattrocchio et al., 2008; Tonni, 2017). These floral changes likely affected strong faunal changes especially for herbivores. Despite a few species with omnivorous diets, such as euphractines (Carlini et al., 2016; Superina & Abba, 2014), or occasional meat consumption in some sloths, such as Darwin's ground sloth (Tejada et al., 2021), xenarthrans were predominantly herbivorous (e.g., Bargo & Vizcaíno, 2008; Gaudin & Croft, 2015; Toledo et al., 2015; Vizcaíno & Loughry, 2008). The high herbivore dominance of xenarthrans from this period thus makes them good indicators of paleoenvironmental change as already highlighted by many studies (e.g., Soibelzon & Tonni, 2009; Soibelzon, 2019; Tonni et al., 1999a, 1999b). This observation is supported by isotopic analyses of the teeth of several herbivores, demonstrating a modification of the herbivorous diet associated with environmental changes induced by climate change (e.g., De Melo França et al., 2015—see below). According to some authors, some xenarthran species were associated with arid environments such as *Neosclerocalyptus paskoensis* and *Scelidotherium bravardi* (Miño-Boilini & Quiñones, 2020; Zurita et al., 2011) while other species to closer environments such as *Pampatherium humboldtii* (Varela et al., 2018) and *Catonyx cuvieri* (Miño-Boilini & Quiñones, 2020) based on their cranial and/or dental anatomy. Following these paleobiological interpretations, the Roth collection at PIMUZ contains a mix of these specialized species likely due to the high concentration of localities found in the northern Pampean Region (Fig. 2). Differences in the diet of the species have been suggested for some taxa, using isotope analysis (De Melo França et al., 2015). For example, *Glyptodon* from latitude 37°S had an exclusively C_3_ grasses consumption while a *Glyptodon* from latitude 32°S had a mixed consumption of C_4_ plants and C_3_ grasses, demonstrating the higher aridity of the southern Pampean Region compared to the north, during the Late Pleistocene (De Melo França et al., 2015).

During the Ensenadan, Roth collection at PIMUZ shows the presence of *Chaetophractus vellerosus*, *Eutatus pascuali*, a great abundance of *Glyptodon munizi*, *Neosclerocalyptus pseudornatus*, *Neosclerocalyptus ornatus*, a representative of *Lestodon*, *Glossotherium robustum*, *Mylodon darwinii*, *Scelidotherium bravardi*, *Catonyx tarijensis*, and *Megatherium americanum* (Fig. 2). During this time span, two mayor glaciations’ events have been reported, the Great Patagonian Glaciation and the Matuyama/Brunhes Glaciation, that may suggest that the animals were inhabitants of mostly arid/open environments. At the end of the Ensenadan and during the Bonaerian, Roth collection at PIMUZ seems to indicate a greater diversity with the appearance in the Pampean Region of a representative of *Tolypeutes*, *Pampatherium humboldtii*, *Pampatherium typum*, *Doedicurus clavicaudatus*, *Neosclerocalyptus gouldi*, *Panochthus intermedius*, *Panochthus tuberculatus*, *Lestodon armatus*, *Archaeomylodon sampedrinensis*, *Scelidotherium leptocephalum*, and *Nothrotherium escrivanense*, although the collection shows a disappearance from the Pampean Region of *Chaetophractus vellerosus*, *Neosclerocalyptus pseudornatus*, and *Neosclerocalyptus ornatus* (Fig. 2). This period is associated with a global warming marked by two peaks of warming highlighted by Marine Isotope Stages, suggesting that the Ensenadan fauna mixed with fauna that inhabited more humid/closed environments. Finally, during the Lujanian, Roth's collection at PIMUZ exhibits a disappearance of much of the Bonaerian fauna and the presence of *Propraopus sulcatus*, *Zaedyus pichiy*, *Eutatus seguini*, *Pampatherium humboldtii*, *Glyptodon reticulatus*, *Neosclerocalyptus paskoensis*, a representative of *Panochthus*, *Glossotherium robustum*, *Mylodon darwinii*, *Scelidotherium leptocephalum*, *Catonyx tarijensis*, and *Catonyx cuvieri* (Fig. 2). The Lujanian is marked by a major cooling notably well marked by the Last Glaciation Maximum and corresponds to the return of an arid/open environment, but with a different faunal assemblage than the Ensenadan, suggesting a faunal replacement following the warming during the Bonaerian (Fig. 2). Through the various climatic and environmental changes occurring during the Pleistocene, the Roth collection at PIMUZ shows changes in faunal assemblages but the collection does not indicate a major loss of diversity. The Roth collection at PIMUZ shows several phases of extinction and appearance of several xenarthran species, evidence that xenarthran species have undergone drastic turnovers in the Pleistocene of the Pampean Region.

Due to the limitation of the relative dating of the specimens and the partial nature of the associated data, especially because this collection was sold to different buyers, I cannot propose a further interpretation of the relationship between the xenarthrans of the Santiago Roth collection of the PIMUZ and paleoenvironmental variation. However, the major faunal trends detected among Pleistocene subdivisions are in agreement with most previous studies (Carlini & Scillato-Yané, 1999; Cione et al., 1999; Soibelzon & Tonni, 2009; Soibelzon et al., 2010; Tonni et al., 1999a, b). A few exceptions are represented in the collection by the presence of *Megatherium* and *Doedicurus* from the Ensenadan/Bonaerian. However, these same taxa are those for which paleontologists call for further revisions of their diversity, a next step for our understanding of the evolution of Pleistocene mammalian faunas from the Pampean Region.

## Conclusions

Since the nineteenth century, many studies have focused on Pleistocene mammals of the Pampean Region to understand the evolution of paleobiodiversity and the Quaternary paleoclimate of southern South America. The evolution of the South American megafauna and its relation with climatic change is a rich subject of investigation. Among their emblematic representatives, xenarthrans are one of the most abundant taxa of South American megafauna. Since the first works of eminent paleontologists, our knowledge on the evolution of Pleistocene xenarthrans of the Pampean Region has been refined, and the group has benefited from a strong taxonomic revision in the last 20 years. In the present study, I investigated one of the largest collections of Pleistocene xenarthrans held in Europe: the Santiago Roth collection of the PIMUZ. I revised 140 xenarthran specimens, leading to the proposal of 114 taxonomic reassignments. This work has allowed to assess the great diversity of xenarthrans present in the collection, the preservation and rarity of many specimens, and to specify their interest for the study of evolutionary, functional, and paleoecological/paleobiogeographical reconstruction. In particular, the faunal assemblages of the xenarthrans from the Roth collection at PIMUZ follow the major paleoenvironmental variations known for the Pleistocene Pampean Region in relation to the Quaternary paleoclimate. Hopefully, this type of investigation will encourage the taxonomic reappraisal of other South American paleontological collections housed in the northern hemisphere.

### Supplementary Information

**Additional file 1: Table S1.** Additional information per specimen. The numbered localities are linked to Fig. 2. Symbol: † Extinct.

**Additional file 2: Appendix S1.** List of references related to Table S1.

## Data Availability

All data related to the expertise work of the xenarthrans of the Santiago Roth collection at the PIMUZ are given in the present publication and in Additional file 1: Table S1. CT-scans data on many of the specimens are becoming available in publications concurrent to the present one.
