# Supplementary material for: Xenarthrans of the collection of Santiago Roth from the Pampean Region of Argentina (Pleistocene), in Zurich, Switzerland
Source: Swiss J Palaeontol. 2023 Mar 28;142(1):3. doi: 10.1186/s13358-023-00265-7 (PMC10049960; doi:10.1186/s13358-023-00265-7)
Supplement: Supplementary file 2 — Additional file 2: Appendix S1. List of references related to Table S1. [file 13358_2023_265_MOESM2_ESM.docx]

**REFERENCES FOR TABLE S1**

Ameghino, F. (1881). *La Antigüedad del Hombre en el Plata 2*. Paris and Buenos Aires: Masson-Igon.

Ameghino, F. (1888). *Rápidas diagnosis de algunos mamíferos fósiles nuevos de la República Argentina*. Buenos Aires: Coni P.E.

Brandoni, D., & Vezzosi, R.I. (2019). *Nothrotheriops* sp. (Mammalia, Xenarthra) from the Late Pleistocene of Argentina: implications for the dispersion of ground sloths during the Great American Biotic Interchange. *Boreas*, *48*(4), 879-890. doi: https://doi.org/10.1111/bor.12401.

Corona, A., Perea, D., & McDonald, H.G. (2013). *Catonyx cuvieri* (Xenarthra, Mylodontidae, Scelidotheriinae) from the late Pleistocene of Uruguay, with comments regarding the systematics of the subfamily. *Journal of Vertebrate Paleontology*, *33*(5), 1214-1225. doi: https://doi.org/10.1080/02724634.2013.764311.

De Iuliis, G.D. (1996). *A systematic review of the Megatheriinae (Mammalia: xenarthramegatheriidae)*. Ottawa: National Library of Canada, Doctoral dissertation.

Desmarest, A.G. (1819). *Nouveau dictionnaire d'histoire naturelle, appliquée aux arts, à l'agriculture, à l'économie rurale et domestique, à la médecine, etc*. (pp. 552). Paris: Chez Deterville, volume 32. doi: https://doi.org/10.5962/bhl.title.20211.

Geoffroy St.-Hilaire, E.I. (1847). Note sur le Genre *Apar*, sur ses Espèces et sur ses Caractères, Établis jusqu´à Présent d´après un Animal Factice. *Revue de Zoologie*, *10*, 135-137.

Gervais, H., Ameghino, F. (1880). Les mammifères fossiles de l'Amérique du Sud (pp. 225). Paris: F. Savy. doi: <https://doi.org/10.5962/bhl.title.61646>

Guth, C. (1961). *La région temporale des Édentés* (pp. 191). Paris: Le Puy, impr. Jeanne d'Arc, Doctoral dissertation.

Houssaye, A., Waskow, K., Hayashi, S., Cornette, R., Lee, A.H., & Hutchinson, J.R. (2016a). Biomechanical evolution of solid bones in large animals: a microanatomical investigation. *Biological Journal of the Linnean Society*, *117*(2), 350-371. doi: https://doi.org/10.1111/bij.12660.

Houssaye, A., Fernandez, V., & Billet, G. (2016b). Hyperspecialization in Some South American Endemic Ungulates Revealed by Long Bone Microstructure. *Journal of Mammalian Evolution*, 23**,** 221-235. doi: https://doi.org/10.1007/s10914-015-9312-y.

Krmpotic, C.M., Carlini, A.A., & Scillato-Yané, G.J. (2009). The species of *Eutatus* (Mammalia, Xenarthra): Assessment, morphology and climate. *Quaternary International*, *210*(1-2), 66-75. doi: https://doi.org/10.1016/j.quaint.2009.06.031.

Lydekker, R. (1887). *Catalogue of the Fossil Mammalia in the British Museum, (Natural History): The group Tillodontia, the orders Sirenia, Cetacea, Edentata, Marsupialia, Monotremata, and Supplement* (pp. 268). London; Printed by order of the Trustees, volume 5. doi: https://doi.org/10.5962/bhl.title.61849.

Lydekker, R. (1895). Contributions to a knowledge of the fossil vertebrates of Argentina. The extinct edentates of Argentina. *Anales del Museo de La Plata, Paleontología Argentina*, *3*, 1-118.

Miño-Boilini, Á.R. (2012). *Sistemática y evolución de los Scelidotheriinae (Xenarthra, Mylodontidae) cuaternarios de la Argentina* (pp. 301). La Plata: Universidad Nacional de La Plata, Doctoral dissertation. doi: https://doi.org/10.35537/10915/24819.

Miño-Boilini, Á.R., Carlini, A.A., & Scillato, G.J. (2014). Revisión sistemática y taxonómica del género *Scelidotherium* owen, 1839 (Xenarthra, Phyllophaga, Mylodontidae). *Revista Brasileira de Paleontologia*, 17(1), 43-58. doi: https://doi.org/10.4072/rbp.2014.1.05.

Miño-Boilini, Á.R., & Zurita, A.E. (2015). Dimorphism in quaternary scelidotheriinae (mammalia, xenarthra, phyllophaga). *Palaeontologia Electronica*, *18*(1.12A), 1-16. doi: http://palaeo-electronica.org/content/2015/1102-dimorphism-in-scelidotheriinae.

Miño-Boilini, Á.R. (2016). Additions to the knowledge of the ground sloth *Catonyx* *tarijensis* (Xenarthra, Pilosa) in the Pleistocene of Argentina. *Paläontologische Zeitschrift*, *90*(1), 173-183. doi: https://doi.org/10.1007/s12542-015-0280-6.

Miño-Boilini, Á.R., & Quiñones, S.I. (2020). Los perezosos Scelidotheriinae (Xenarthra, Folivora): taxonomía, biocronología y biogeografía. *Revista del Museo Argentino de Ciencias Naturales*, *22*(2), 201-218.

Owen, R. (1839a). Description of a tooth and part of the skeleton of the *Glyptodon*, a large quadruped of the edentate order, to which belongs the tessellated bony armour figured by Mr Clift in his memoir on the remains of the *Megatherium*, brought to England by Sir Woodbine Parish, F.G.S. *Proceedings of the Geological Society of London*, *3*, 108–113.

Owen, R. (1839b). *Zoology of the Voyage of the Beagle, under the command of Captain Fitzroy, R.N., during the years 1832-1826. Part 1, Fossil Mammalia* (pp. 111). London: Smith, elder and Co.

Owen, R. (1842). *Description of the skeleton of and extinct gigantic sloth, Mylodon robustus Owen, with observations on the osteology, natural affinities, and probable habits of the megatherioid quadrupeds in general*. London: Taylor, R. & J.

Owen, R. (1845). *Descriptive and illustrated catalogue of the fossil organic remains of Mammalia and Aves contained in the Museum of the Royal College of Surgeons of London* (pp. 391). London: Royal College of Surgeons of England. doi: https://doi.org/10.5962/bhl.title.105383.

Pouchet, G. (1866). Contribution a l'anatomie des édentés. *Journal de L'anatomie et de la Physiologie*, *3*, 337-353.

Roth, S. (1889). Fossiles de La Pampa, Amérique du Sud, collectionnés par Santiago Roth. Catalogue n°5 (pp. 16). Zurich: Jean Meyer.

Schulthess, B. (1920). *Beiträge zur kenntnis der Xenarthra auf grund der Santiago Roth'schen sammlung des Zoologischen museums der Universität Zürich.* (pp. 119). Zurich: Albert Kundig, volume 44.

Straehl, F.R., Scheyer, T.M., Forasiepi, A.M., MacPhee, R.D., & Sánchez-Villagra, M.R. (2013). Evolutionary patterns of bone histology and bone compactness in xenarthran mammal long bones. *PLoS One*, *8*(7), e69275. doi: https://doi.org/10.1371/journal.pone.0069275.

Vezzosi, R.I., Brandoni, D., Brunetto, E., & Zalazar, M.C. (2019). New remains of Nothrotheriinae (Mammalia, Xenarthra) from Late Pleistocene fluvial deposits of Northern Pampa (Santa Fe Province, Argentina). *Journal of South American Earth Sciences*, *89*, 47-54. doi: https://doi.org/10.1016/j.jsames.2018.11.004.
